# Intermediate insights: tracing trematodes infecting amphibians via their first intermediate snail hosts

**DOI:** 10.1186/s13071-025-06920-x

**Published:** 2025-07-15

**Authors:** Annabell Hüsken, Jessica Schwelm, Sonja Rückert, Bernd Sures

**Affiliations:** 1 Aquatic Ecology, University of Duisburg-Essen, Essen, Germany; 2 Research Center One Health Ruhr, Research Alliance Ruhr, University of Duisburg-Essen, Essen, Germany; 3 Centre for Water and Environmental Research, University of Duisburg-Essen, Essen, Germany; 4 Water Research Group, Unit for Environmental Sciences and Management, North-West University, Potchefstroom, South Africa; 5 Eukaryotic Microbiology, University of Duisburg-Essen, Essen, Germany; 6 Centre for Conservation and Restoration Science, Edinburgh Napier University, Edinburgh, UK

**Keywords:** Amphibia, Digenea, Trematoda, Freshwater snails

## Abstract

**Background:**

Amphibians are a prime example of the global biodiversity crisis, as they represent the most threatened group of vertebrates. Yet, amphibian macroparasites remain one of the most poorly described groups of parasites, with the majority of species lacking comprehensive morphological, molecular, or ecological data. Among these, digenean trematodes constitute a dominant group and feature multi-host life cycles. This study examines the first intermediate hosts of trematodes, aquatic gastropods, to assess the occurrence, prevalence, and seasonality of amphibian trematodes.

**Methods:**

A total of 5362 snails from five families (Bithyniidae, Hydrobiidae, Lymnaeidae, Physidae, and Planorbidae) were collected in three stream systems in North Rhine-Westphalia, Germany and investigated for trematode infections. Detailed information on amphibian-infecting trematode cercarial morphology and measurements were provided via light microscopy and scanning electron microscopy (SEM). Comprehensive molecular analyses were conducted and novel sequences generated for multiple genetic markers (28S rDNA, ITS1-5.8S-ITS2, ITS2, *cox*1, and *nad*1).

**Results:**

Three trematode taxa infecting amphibians as second intermediate and/or definitive hosts (*Lecithopyge*, *Cephalogonimus*, and *Opisthioglyphe*) were identified exclusively from lymnaeid snail hosts. A total of 79 novel sequences were generated for 21 trematode isolates. Phylogenetic analyses based on 28S and ITS1-5.8S-ITS2 sequences resulted in concordant taxonomies. Distinct seasonal infection patterns allowed for insights into the species’ life cycles.

**Conclusions:**

Our findings highlight significant gaps in the knowledge of amphibian macroparasites and underline the value of studying cercariae occurrence in snail intermediate hosts as a method for monitoring amphibian trematode biodiversity without affecting amphibian populations.

**Graphical Abstract:**

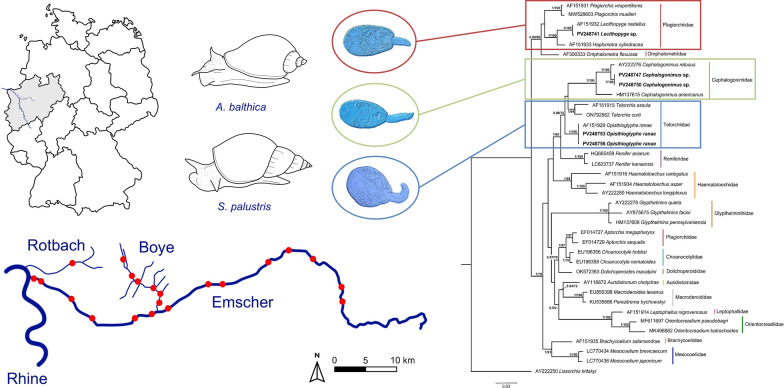

**Supplementary Information:**

The online version contains supplementary material available at 10.1186/s13071-025-06920-x.

## Background

Global biodiversity is experiencing significant declines. Species are disappearing at an unprecedented rate, which is increasingly accelerated by human activity [1–3]. Among vertebrates, amphibians serve as a key example of the biodiversity crisis, representing the most threatened class, with major population declines and 40% of species at risk of extinction [4]. As ectotherms with partially aquatic life cycles, amphibians are particularly sensitive to environmental changes and therefore vulnerable to a range of stressors, including habitat loss, climate change, invasive species, and emerging infectious diseases [5–7]. Among the latter, particularly directly transmitted pathogens such as ranaviruses or the chytrid fungi *Batrachochytrium dendrobatidis* (Bd) and *B. salamandrivorans* (Bsal) have been intensively studied for their role in population declines and species extinctions [8–11].

In contrast, macroparasites have received less attention as pathogens of amphibians, often assumed to exert less pathogenic impact [10, 12]. Existing research has focused primarily on taxa with evident impact on host fitness and survival, such as the nematode genus *Rhabdias*, which parasitizes the lungs of amphibians [13], and the trematode *Ribeiroia ondatrae*, which causes severe limb deformations and increased mortality in amphibian hosts [14]. However, there is growing recognition that a comprehensive understanding of all parasites within ecosystems is pivotal, as infections not only induce pathology but also reduce host fitness by interacting with and potentially enhancing other pathogens and environmental stressors [15–17]. Despite this, amphibian macroparasites remain among the most poorly described parasite groups [18], and regulatory and ethical constraints limit invasive sampling of protected amphibian hosts. The few studies published focus on individual host or parasite species and seldom incorporate molecular analyses or multiple life cycle stages (e.g., [19–21] and [22–24]). Consequently, morphological and genetic data remain sparse for many amphibian parasite taxa and their life stages, impeding accurate species delimitation, phylogenetic inference, reconstruction of life cycles, and evaluation of host specificity.

**Fig. 1:**
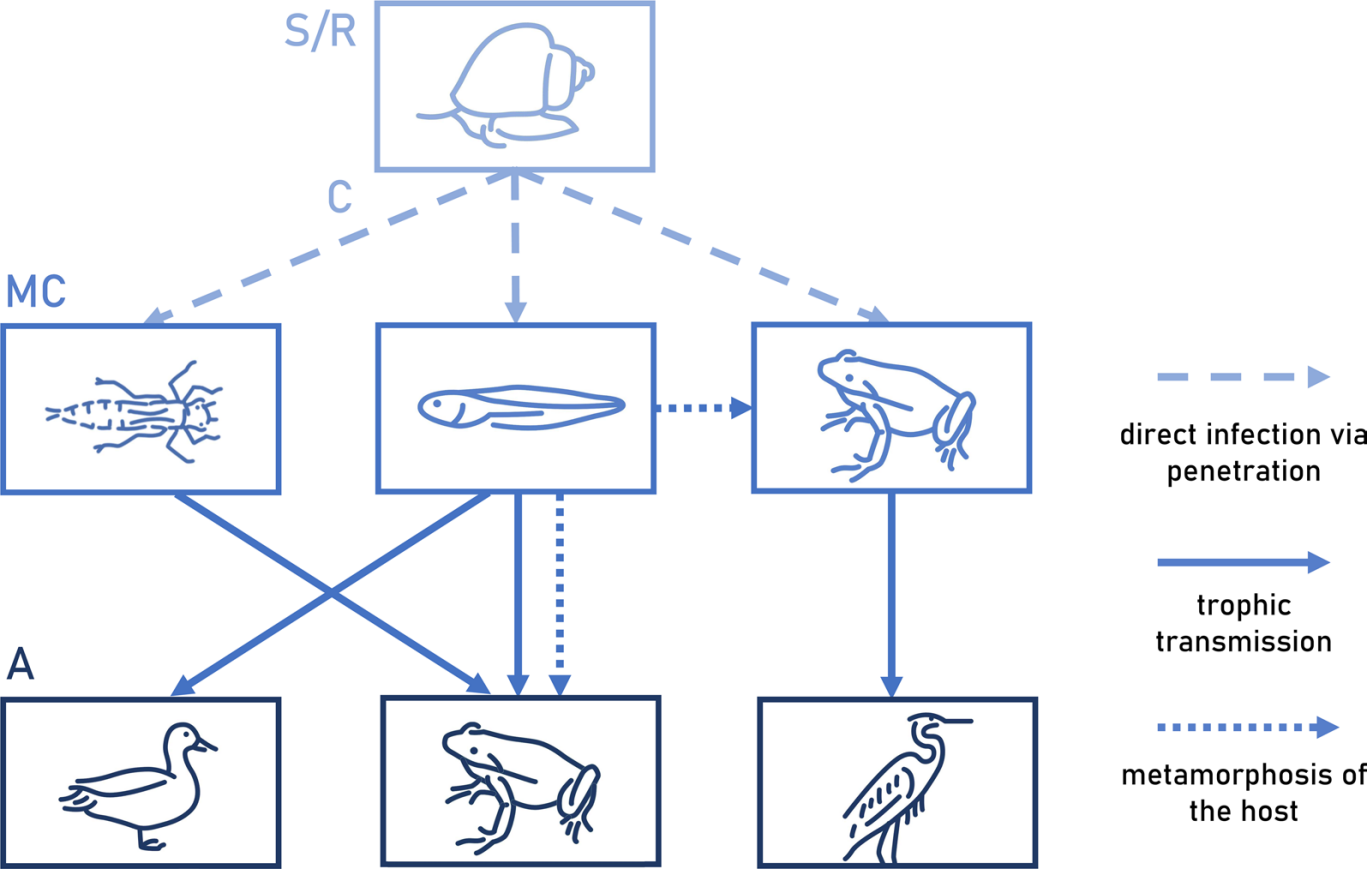
Schematic diagram of transmission pathways involving amphibians as second intermediate and/or definitive hosts in the life cycles of trematodes. Trematode life cycle stages: S/R, sporocysts/rediae; C, cercariae; MC, metacercariae; A, sexual adults

A promising approach to fill these gaps lies in investigating the occurrence and prevalence of macroparasites by targeting life cycle stages in the respective intermediate hosts. Digenean trematodes are among the most prevalent macroparasites of amphibians and feature multi-host life cycles, typically with two intermediate hosts, the first of which is usually an aquatic gastropod [12, 25, 26]. Depending on the trematode species, amphibians may function either as second intermediate hosts (e.g., *Cathaemasia* spp.) or definitive hosts (e.g., *Haematoloechus* spp.) in the life cycle (Fig. 1) [27, 28]. As second intermediate hosts, amphibians usually become infected early in their development, following the emission of free-living cercariae from first intermediate hosts. The cercariae penetrate the skin and encyst as metacercariae in various host tissues. As definitive hosts, amphibians become infected by ingesting arthropod or amphibian intermediate hosts. However, it has also been demonstrated that the same infected amphibian can function as both second intermediate and definitive host, for instance, through ingestion of metacercariae in their shed skin or when metacercariae hatch and migrate to the intestine, where they mature into sexual adult trematodes [12, 29].

By shifting the focus to first intermediate gastropod hosts, we highlight a noninvasive approach to monitor the diversity, distribution, and transmission dynamics of trematode species known to infect amphibians (hereafter “amphibian trematodes”) without impacting protected amphibian populations. In this study, we integrate detailed morphological and molecular analyses of larval amphibian trematodes recovered from gastropod hosts, offering new insights into their local occurrence and life cycle progression. Our findings contribute to a more complete understanding of amphibian trematode biodiversity and provide novel data to complement existing and future noninvasive studies on amphibian trematodes.

## Methods

### Sample collection

**Fig. 2:**
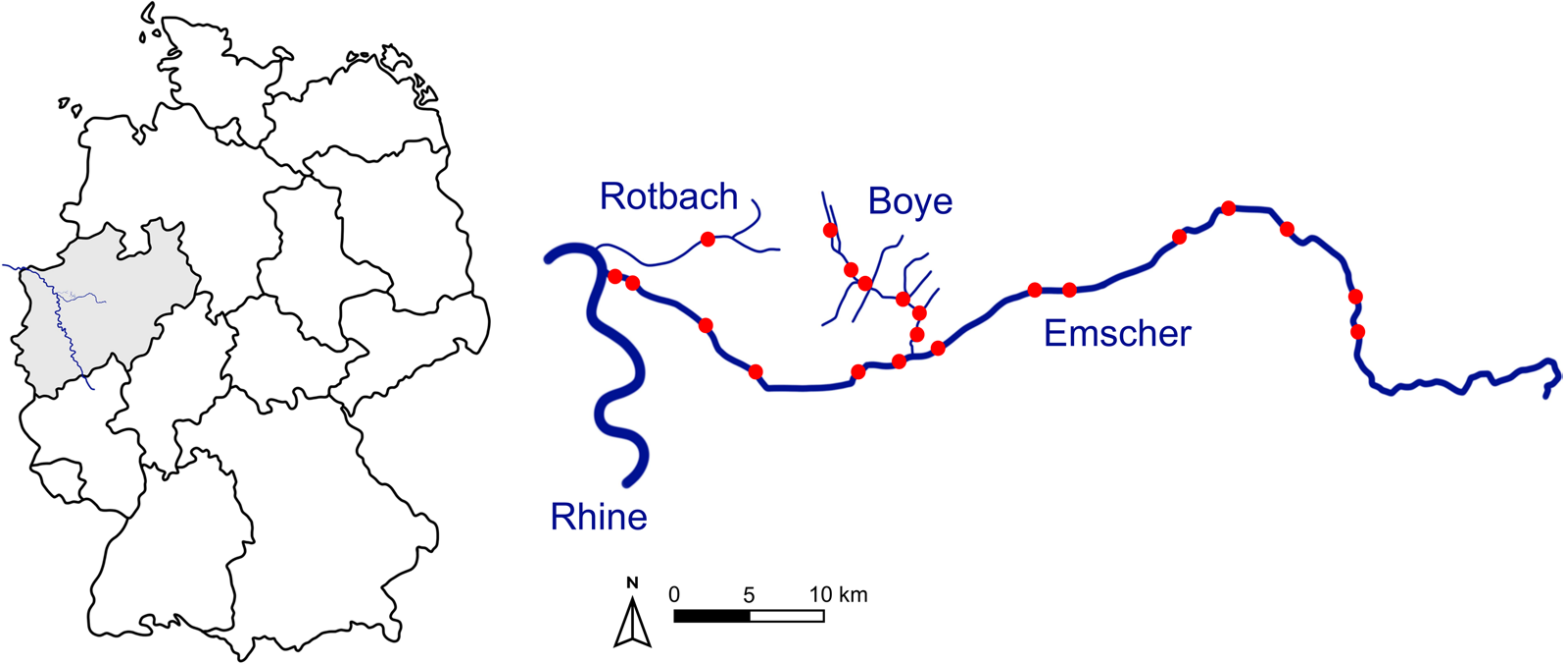
Map of Germany and the catchment area of the Emscher, Boye, and Rotbach. Red dots indicate sampling locations

Snails for the present study were collected from several locations at three rivers (Boye, Emscher, and Rotbach) in North Rhine-Westphalia, Germany (Fig. 2) as part of a large field study associated with the Collaborative Research Centre RESIST (https://sfb-resist.de). Monthly samplings were conducted along the Boye river from May 2023 to January 2025. Seasonal samplings were conducted in spring (March), summer (July), and autumn (October) 2024 at the Emscher river. Additional snails were sampled at the Rotbach river in spring (May) 2024. A total of 5362 snails from the families Bithyniidae (247 snails), Hydrobiidae (22), Lymnaeidae (4728), Physidae (71), and Planorbidae (294) were collected by hand or with sieves from sediments, submerged vegetation, and stones along the riverbank or river bed. In the laboratory, snails were identified to species-level using the identification key by Glöer [30]. The snails were then placed individually in beakers filled with filtered water from the respective sampling site and exposed to a light source for 3 consecutive days to induce cercarial emergence from infected hosts. Each beaker was screened daily under a stereomicroscope for patent trematode infections. Snails collected from the Emscher river and Rotbach river, including specimens for which cercarial emergence was not observed, were dissected and examined for intramolluscan trematode stages such as sporocysts or rediae. Snails collected during monthly samplings along the Boye river were released back to their respective habitats after 3 days as part of an ongoing mark–release–recapture project, where we solely focus on patent infections with cercarial emergence.

### Morphological analyses

Morphological identification of cercariae was conducted for live specimens under an Olympus BX51 microscope using identification keys [31, 32]. For infections where specimens could not directly be identified to species-level, a preliminary identification was made to family- or genus-level and subsequently refined with molecular analyses. Trematode taxa known to infect amphibians (78 infected snails in total) were subjected to further morphological and molecular analyses. To document their cercarial morphology in detail, high-resolution images of live individuals of each species were captured with an Olympus UC30 digital camera attached to the light microscope. Measurements for each specimen were obtained with the assistance of ImageJ image analysis software [33]. All measurements are provided in micrometres (µm) and are presented as a range, followed by the mean value in parentheses. Drawings of the cercariae were based on combined information from several microphotographs taken of live specimens.

For scanning electron microscopy (SEM), glutaraldehyde-fixed cercariae were transferred to a multi-well plate and washed ten times in distilled water. A sheet of filter paper was attached to the inside of the plate lid and soaked with 4% (w/v) osmium tetroxide. The lid was placed on the multi-well plate, and the specimens were fixed in osmium tetroxide vapor in the dark for 1 h. Subsequently, five drops of 4% (w/v) osmium tetroxide were added directly to the wells, and the samples were fixed again in the dark for 1 h. The samples were washed ten times with distilled water and placed in microporous specimen containers (pore size 78 µm), dehydrated through a graded ethanol series, and then critical-point dried using liquid CO_2_ (Leica EM CPD 300). The samples were placed on stubs, sputter-coated with platinum/palladium (5 µm, Leica EM ACE 600), and subsequently analyzed under the scanning electron microscope (Zeiss Crossbeam 540). SEM analyses were used for description of cercarial surface morphology; however, SEM measurements were not generated as the sample preparation induces shrinkage of specimens.

### Molecular analyses

Trematode material obtained from all infected hosts was preserved in molecular-grade (96%) ethanol for molecular analyses. Total genomic DNA was extracted using a modified salt precipitation protocol after Grabner et al. [34], as described in Schwelm et al. [35]. For all isolates, amplification of the D1–D3 domains of 28S rDNA was conducted using universal trematode primers digl2 and 1500R (Table 1). The polymerase chain reaction (PCR) products were purified and Sanger sequenced at Microsynth Seqlab (Germany) using the forward primer. All sequences were checked in Geneious Prime (2024.0.5; Biomatters, Auckland, New Zealand) and attributed a (preliminary) species ID by comparison against the National Center for Biotechnology Information (NCBI) nucleotide database using the Basic Local Alignment Search Tool (BLAST; www.ncbi.nih.gov/BLAST/).

**Table 1:** PCR primers for gene fragments used in this study

Gene fragment	Primer name	Fragment length	Nucleotide sequence (5′–3′)	Source
28S	digl2	~1200 base pairs (bp)	AAG CAT ATC ACT AAG CGG	[81]
	1500R		GCT ATC CTG AGG GAA ACT TCG	[81]
	300F_a_		CAA GTA CCG TGA GGG AAA GTT G	[82]
	EC–D2_a_		CCT TGG TCC GTG TTT CAA GAC GGG	[83]
ITS1-5.8S-ITS2	D1	~600 bp	AGG AAT TCC TGG TAA GTG CAA	[84]
	D2		CGT TAC TGA GGG AAT CCT GGT	[84]
ITS2	3S	~400 bp	GGT ACC GGT GGA TCA CGT GGC TAG TG	[85]
	ITS2		CCT GGT TAG TTT CTT TTC CTC CGC	[86]
*cox*1	JB3	~400 bp	TTT TTT GGG CAT CCT GAG GTT TAT	[87]
	JB4.5		TAA AGA AAG AAC ATA ATG AAA ATG	[87]
	Dice1F	~800 bp	ATT AAC CCT CAC TAA ATT WCN TTR GAT CAT AAG	[88]
	Dice14R		TAA TAC GAC TCA CTA TAC CHA CMR TAA ACA TAT GAT G	[88]
*nad*1	NDJ11	~500 bp	AGA TTC GTA AGG GGC CTA ATA	[89]
	NDJ2a		CTT CAG CCT CAG CAT AAT	[89]

_a_Sequencing primer

On the basis of recommendations presented in Blasco-Costa et al. [36], three to five isolates for every host–parasite–location combination of amphibian-infecting trematodes were subjected to further molecular analyses. In addition to the 28S rDNA, we sequenced the internal transcribed spacer region, two slightly overlapping sections of the mitochondrial cytochrome c oxidase subunit 1 gene (*cox*1) commonly used for trematode species delimitation, and a section of the mitochondrial nicotinamide adenine dinucleotide dehydrogenase subunit 1 (*nad*1) (Table 1). All PCR reactions were performed using a 20 µL assay with 10 µL of Dream-Taq^™^ Hot Start Green PCR Master Mix (Thermo Fisher Scientific, Waltham, MA, USA), 1.6 µL of each primer, 4.8 µL of nuclease-free water, and 2 µL of DNA per reaction. Thermocycling conditions for all primers followed the protocols in the source papers (Table 1). PCR products for amphibian trematode isolates were purified and Sanger sequenced from both ends at Microsynth Seqlab (Germany) using the respective PCR primers. The sequences were assembled and edited using Geneious Prime (2024.0.5; Biomatters, Auckland, New Zealand) and deposited in GenBank with the corresponding accession numbers (Table 2). Mitochondrial sequences were checked for the amplification of pseudogenes and annotated using the trematode mitochondrial code (Translation Table 21; https://www.ncbi.nlm.nih.gov/Taxonomy/Utils/wprintgc.cgi#SG21) [37, 38]. The sequences were aligned with selected sequences deposited in GenBank using MUSCLE 5.1 algorithm implemented in Geneious Prime [39]. Owing to the scarcity of within-family reference sequences with significant overlap for the newly generated *cox*1 and *nad*1 sequences, phylogenetic analyses were focused on rDNA markers 28S and ITS1-5.8S-ITS2. The alignments included taxa from the families Cephalogonimidae, Plagiorchiidae, and Telorchiidae, as well as additional representative sequences of other families within the superfamily Plagiorchioidea, provided that a sequence was available for the respective family in GenBank. If possible, we prioritized sequences derived from sexual adult trematodes isolated from definitive hosts. The resulting 28S rDNA alignment (1151 nucleotides (nt)) comprised 32 taxa. The internal transcribed spacer (ITS) alignment (682 nt) included 17 taxa. As all newly generated 28S and ITS1-5.8S-ITS2 sequences were identical within species, only one representative sequence per species for each sampling location and host species was included in the alignments. Sequences of *Lissorchis kritskyi* (Lissorchiidae) from the related superfamily Monorchioidea were used as outgroups. A full list of sequences used for both alignments is provided in Additional File 1: Supplementary Table S1. Phylogenetic trees were constructed using maximum likelihood (ML) and Bayesian inference (BI) analyses. Maximum likelihood trees were constructed using IQ-TREE version 2.3.6 [40] with non-parametric bootstrap validation based on 1000 pseudoreplicates. The best-fitting evolutionary models for ML analyses were selected on the basis of the Akaike information criterion (AIC) in ModelFinder [41]. These were TVM + F + I + G4 for the 28S alignment and TVM + F + I + R2 for the ITS1-5.8S-ITS2 alignment. The BI analyses were performed using MrBayes software version 3.2.7 [42]. The best-fitting evolutionary model for BI analyses was selected using the AIC in MrModelTest version 2.4 [43], which was GTR + I + G for both alignments. Markov chain Monte Carlo chains were run for 10,000,000 generations, log-likelihood scores were plotted to estimate burn-in, and only the final 75% of trees were used to produce the consensus trees. FigTree version 1.4.4 software (http://tree.bio.ed.ac.uk/software/figtree/) was used for tree visualization. Uncorrected *p*-distances between sequences for each genetic marker were calculated using MEGA version 11 [44] and provided in Additional File 2: Supplementary Tables S2–S7.

**Table 2:** Accession numbers of novel sequences generated in this study

Taxon	Isolates_a_	GenBank accession no.					
		28S	ITS1-5.8S-ITS2	ITS2	*cox*1 (Dice1F/Dice14R)	*cox*1 (JB3/JB4.5)	*nad*1
*Lecithopyge* sp.	B2393, B2428, B2442, B2458, B2667, B2671, B2898	PV248741–PV248746	PV252985–PV252991	PV248788–PV248794	PV605625–PV605630	PV605649–PV605654	PV602038–PV602039
*Cephalogonimus* sp.	R4–R6, E106, E235, E243, E259	PV248747–PV248752	PV252992–PV252994	PV248795–PV248800	PV605631 –PV605635, PV605637	PV605655–PV605658	PV602040–PV602041
*Opisthioglyphe ranae*	E240, E564, E566, E663, E670	PV248753–PV248756	PV252995–PV252995	PV248801–PV248804	PV605636, PV605639–PV605642		

_a_Number of isolates submitted to GenBank may be lower than the total number of isolates, as only sequences with > 95% high-quality base calls were selected for submission

## Results

In total, 25 trematode taxa were identified (Additional File 3: Supplementary Table S8), including three distinct genetic lineages known to infect amphibians as second intermediate and/or definitive hosts, belonging to the families Cephalogonimidae, Plagiorchiidae, and Telorchiidae. These lineages were exclusively detected in lymnaeid snails and distributed across the three sampling sites. The majority of infections were found in *Ampullaceana balthica* (formerly *Radix balthica*), with a single infection in *Stagnicola palustris*. In addition, we identified *Echinoparyphium recurvatum* in four snail species (*A. balthica*, *Lymnaea stagnalis*, *S. palustris*, and *Planorbis carinatus*). Although metacercariae of this trematode species have occasionally been reported from amphibians, it is primarily associated with molluscs as second intermediate hosts, and its relevance to amphibian infections remains ambiguous (see Discussion section on *E. recurvatum*). Therefore, our morphological and molecular analyses focus on the three abovementioned lineages.

### Molecular identification of amphibian trematodes

**Fig. 3:**
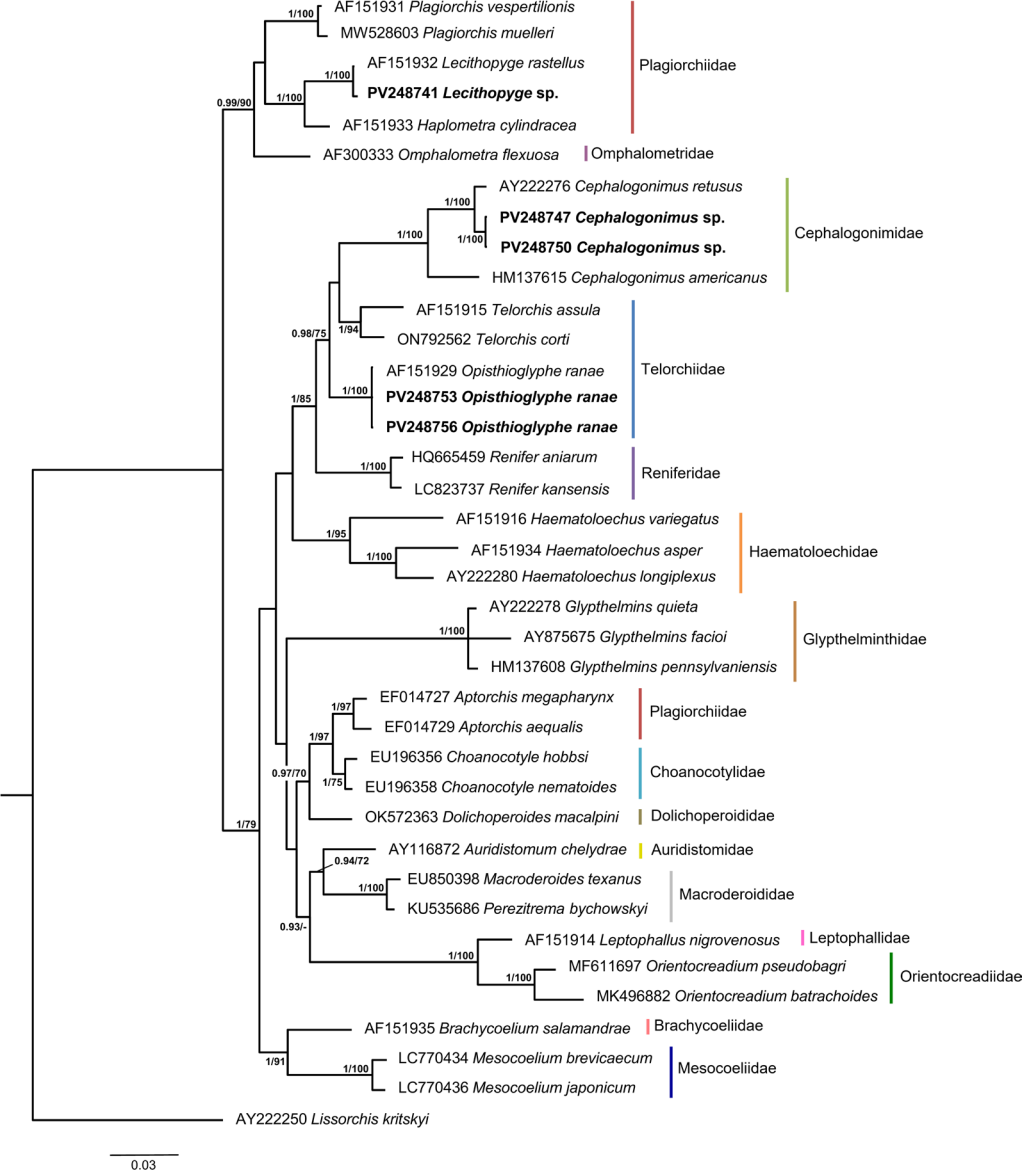
Bayesian inference (BI) phylogram based on partial 28S rDNA sequences for selected species of the superfamily Plagiorchioidea. Node support is indicated by posterior probabilities from BI and bootstrap values from maximum likelihood analysis (ML), respectively. Only values > 0.90 (BI) and > 70 (ML) are displayed. Scale bar indicates the expected number of substitutions per site. Sequences generated in this study are presented in bold

**Fig. 4:**
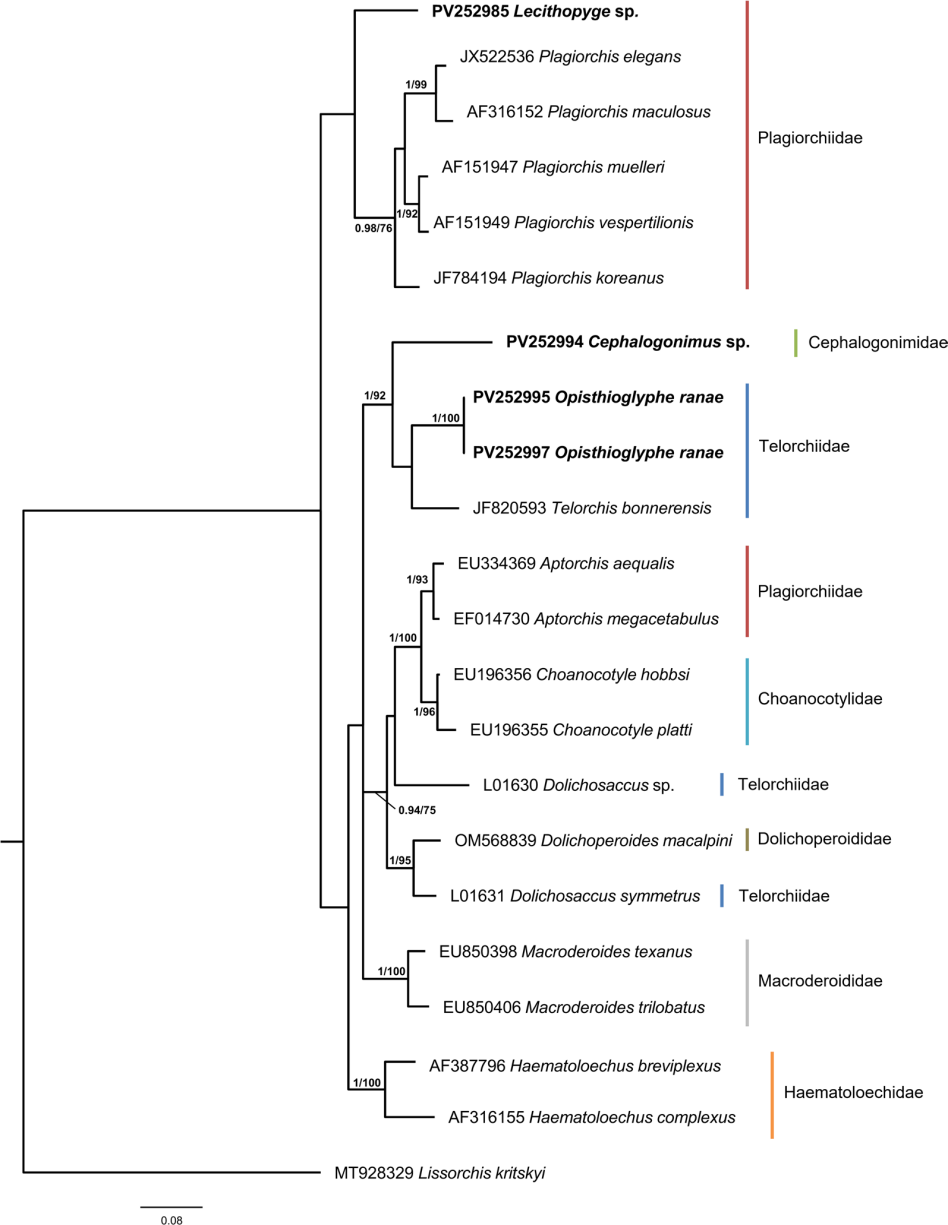
Bayesian inference (BI) phylogram based on ITS1-5.8S-ITS2 sequences for selected species of the superfamily Plagiorchioidea. Node support is indicated by posterior probabilities from BI and bootstrap values from maximum likelihood analysis (ML), respectively. Only values > 0.90 (BI) and > 70 (ML) are displayed. Scale bar indicates the expected number of substitutions per site. Sequences generated in this study are presented in bold

We generated 79 novel sequences for 21 isolates of the three identified trematode lineages known to infect amphibians: sequences of the partial 28S rDNA (16 sequences), ITS1-5.8S-ITS2 (14), and ITS2 (17) regions as well as *cox*1 (28) and *nad*1 (4) regions. The BI and ML phylogenetic analyses based on the newly generated 28S rDNA and ITS1-5.8S-ITS2 sequences resulted in trees with similar topologies (Figs. 3, 4). Notably, the families Plagiorchiidae and Telorchiidae did not form monophyletic clades in either tree.

For the isolates B2393–B2898, the following sequences were generated: 28S (1254 nt), ITS1-5.8S-ITS2 (1114 nt), ITS2 (457 nt), *cox*1 (341 nt and 750 nt), and *nad*1 (456 nt). Both the 28S and the ITS1-5.8S-ITS2 analyses placed the isolates within the family Plagiorchiidae. The 28S rDNA sequences clustered with *Lecithopyge rastellus* (AF151932) ex *Bombina variegata* from Ukraine with strong support (0.2%, 2 nt difference). In the ITS1-5.8S-ITS2 phylogenetic analysis, the isolates were placed basal to representatives of the genus *Plagiorchis*. BLAST analyses based on *cox*1 and *nad*1 genetic markers revealed no closely related sequences for these genetic markers in GenBank, which would be needed to further refine species identification. On the basis of these molecular data, the isolates were identified as *Lecithopyge* sp.

For the isolates R4–R6, E106–E235, and E243–E259, the following sequences were retrieved: 28S (1070 nt), ITS1-5.8S-ITS2 (1356 nt), ITS2 (417 nt), *cox*1 (341 nt and 750 nt), and *nad*1 (456 nt). The 28S sequences clustered within the family Cephalogonimidae with *Cephalogonimus* *retusus* (AY222276) ex *Pelophylax ridibundus* from Bulgaria (0.9%, 10 nt difference) and *C. americanus* (HM137615) ex *Ambystoma velasci* from Mexico (4.2%, 46 nt difference). No related ITS sequences of the family Cephalogonimidae were available in GenBank. In the ITS1-5.8S-ITS2 phylogenetic tree, the isolates grouped basal to a cluster of the family Telorchiidae. The *cox*1 sequences generated with markers JB3 and JB4.5 were identical to each other and differed by 12.3% (42 nt) from *C. americanus* (HM137633) ex *A. velasci* from Mexico. For the *nad*1 genetic marker, no closely related sequences were present in GenBank. On the basis of these data, the isolates were identified as *Cephalogonimus* sp.

For the isolates E240 and E564–E670, the following sequences were retrieved: 28S (1252 nt), ITS1-5.8S-ITS2 (1319 nt), ITS2 (372 nt), and *cox*1 (750 nt). The 28S sequences were identical to *Opisthioglyphe ranae* (AF151929) ex *Rana arvalis* from Ukraine and clustered closely with two representatives of the genus *Telorchis* within the family Telorchiidae. In the ITS phylogenetic analysis, the sequences clustered closest with *T. bonnerensis*. A BLAST analysis based on *cox*1 sequences generated with primer pair Dice1F and Dice14R in GenBank revealed a sequence of *O. ranae* (MW600276) metacercariae from *Dreissena polymorpha* with high similarity to the isolates (0.53–2.14%, 4–16 nt difference). No *nad*1 sequences were retrieved for these isolates. On the basis of these data, the isolates were identified as *O. ranae*.

### Morphological descriptions

**Plagiorchiidae**

*Lecithopyge* sp.

First intermediate host: *Ampullaceana balthica* (Gastropoda, Lymnaeidae).

Location: Boye, Germany.

Prevalence: 1.5% (48 of 3100 snails).

Representative DNA sequences: PV248741–746 (28S); PV252985–991 (ITS1-5.8S-ITS2); PV248788–794 (ITS2); PV605625–630 and PV605649–654 (*cox*1); and PV602038–039 (*nad*1).

**Fig. 5:**
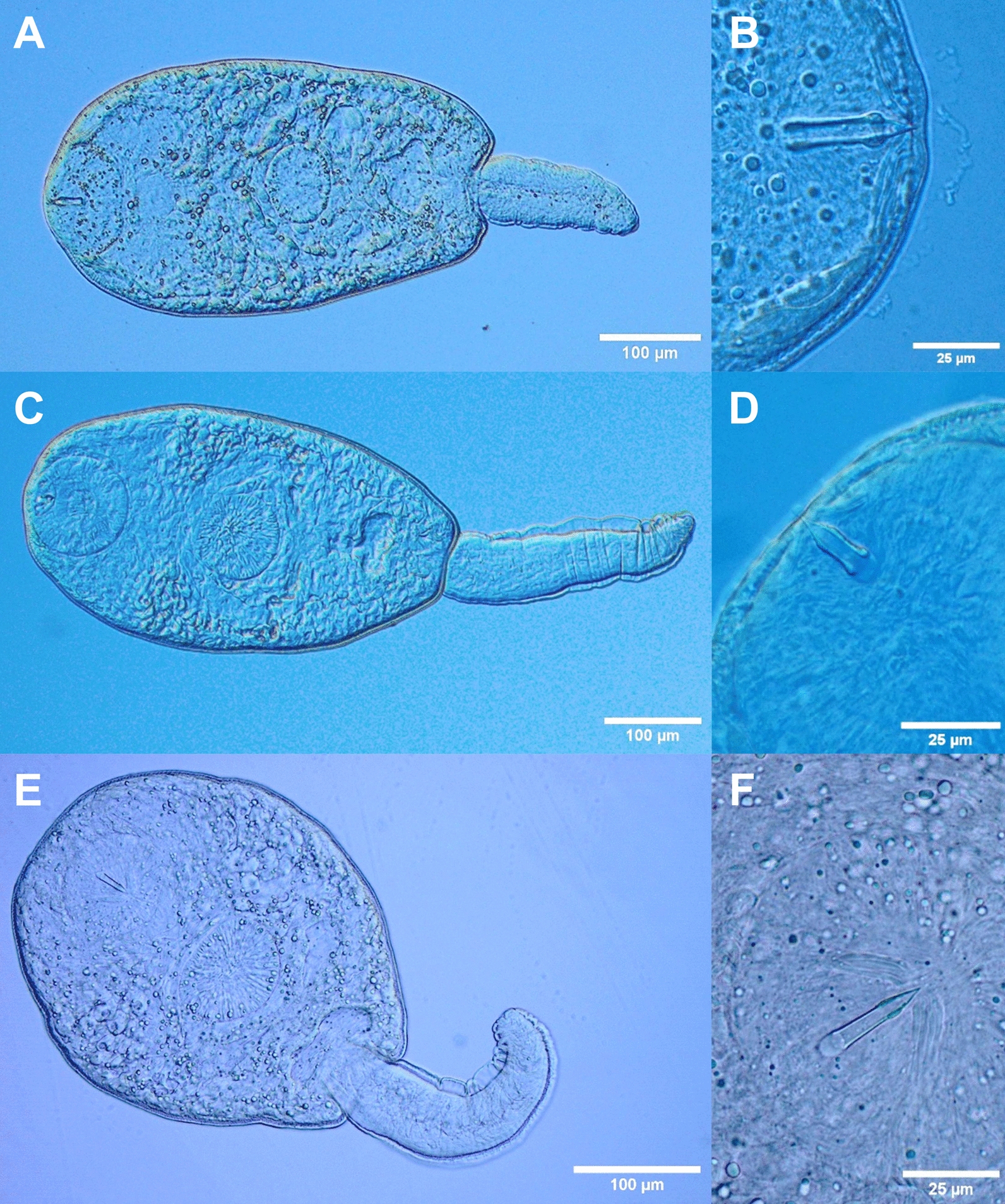
Light micrographs of live cercariae. **A** *Lecithopyge* sp.; **B** *Lecithopyge* sp., stylet; **C** *Cephalogonimus* sp.; **D** *Cephalogonimus* sp., stylet; **E** *Opisthioglyphe ranae*; **F** *O. ranae*, stylet

**Fig. 6:**
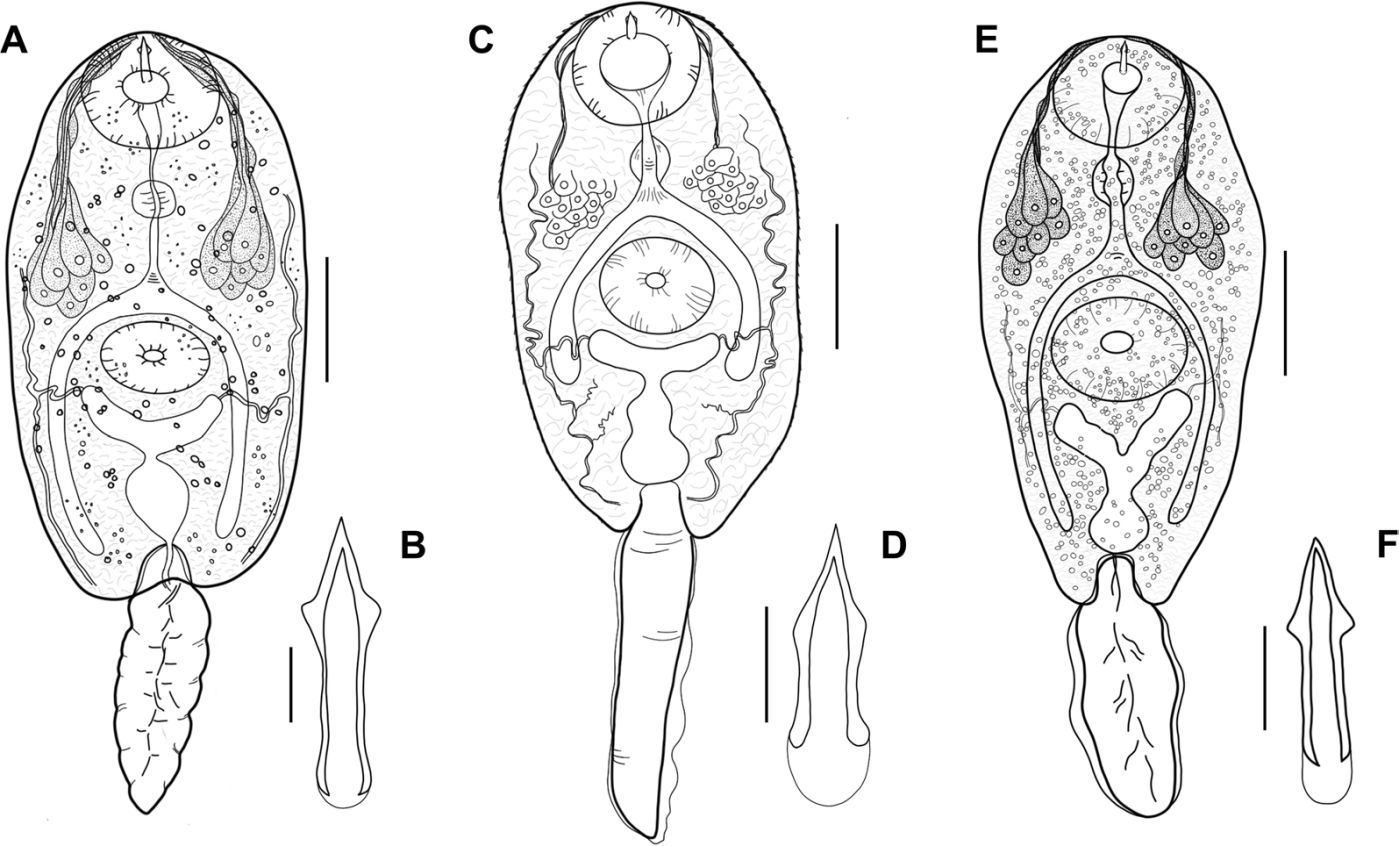
Morphology of trematode cercariae. **A**, **B** *Lecithopyge* sp. (**A** total view; **B** stylet); **C**, **D** *Cephalogonimus* sp. (**C** total view; **D** stylet); **E**, **F** *Opisthioglyphe ranae* (**E** total view; **F** stylet). Scale bars: **A**, **C**, **E**, 100 µm; **B**, **D**, **F**, 10 µm

**Fig. 7:**
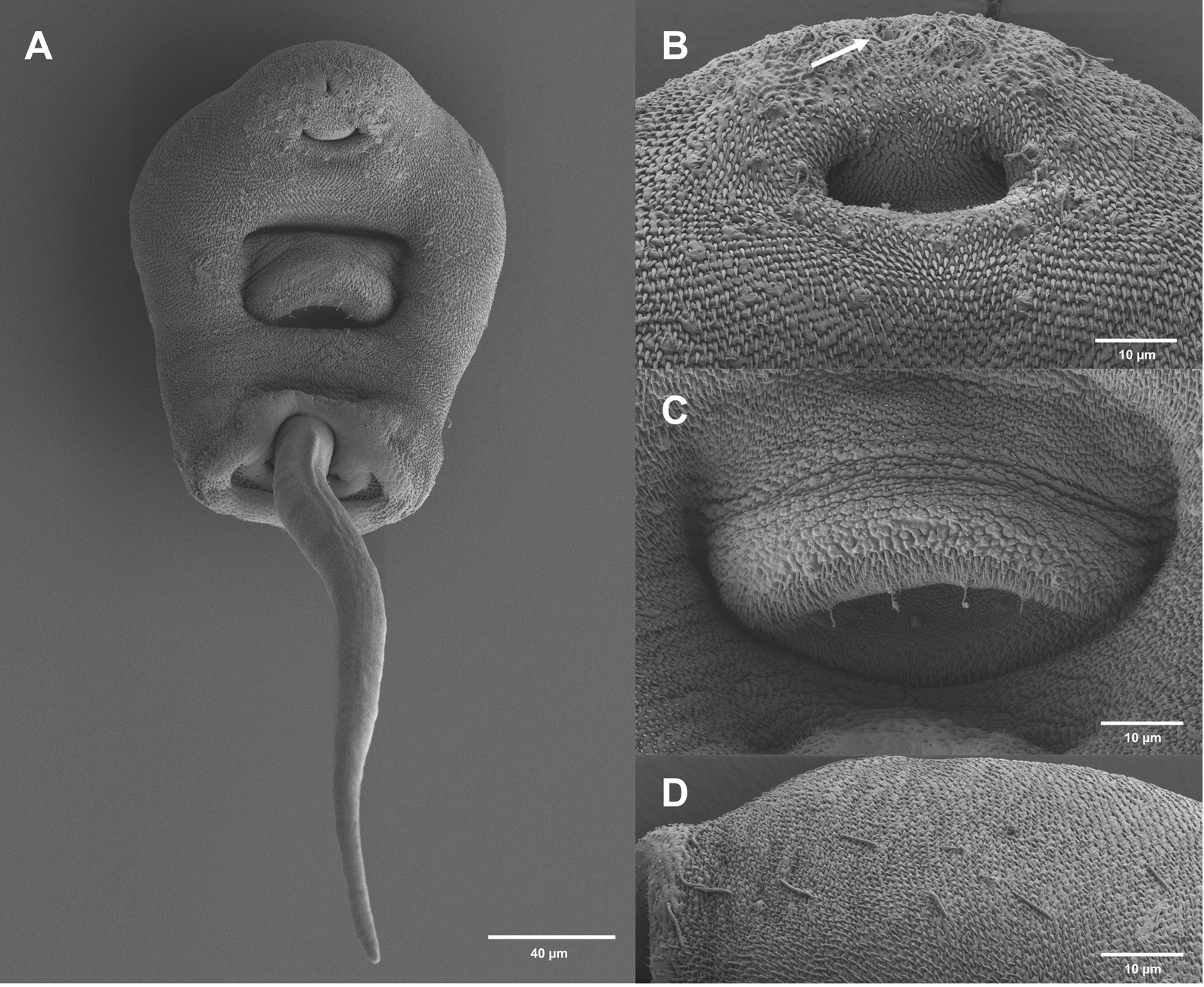
Surface morphology of *Lecithopyge* sp. (SEM). **A** cercarial body, ventral view; **B** stylet opening (white arrow) and oral sucker with sensorial bristles and papillae; **C** ventral sucker with small dome-shaped papillae and sensorial bristles; **D** dorsolateral posterior view, papillae with long sensilla

Cercariae (Figs. 5A, B; 6A, B; 7):

Descriptions and measurements based on ten live specimens and SEM analyses are shown below.

Dark body, elongate oval, maximum width just anterior to ventral sucker; 406–522 $\times$ 183–254 (452 $\times$ 228). Tail simple, short, 140–205 (168) long, 33–45% (37%)  of body length, 40–69 (59) wide at base. Tail without fin fold. Oral sucker well-developed, muscular, subspherical, 89–102 $\times$ 91–109 (94 $\times$ 100). Stylet at anterior level of mouth opening; large, robust, sharply-pointed, 35–41 (38) long. Incomplete basis with thickening, 7–9 (8) wide at base and 6–7 (6) wide above base. Well-developed anterior lateral thickening, 9–11 (10) wide. Stylet width at anterior thickening 22–30% (25%) of stylet length. Ventral sucker subspherical, post-equatorial, 65–73 $\times$ 74–82 (69 $\times$ 77). Ventral sucker smaller than oral sucker, width ratio 1:0.69–0.84 (1:0.77). Prepharynx short, pharynx feebly muscular, indistinct, rounded to oval, 40–48 $\times$ 38–47 (44 $\times$ 43). Oesophagus short, narrow, intestinal bifurcation close to ventral sucker. Caeca reaching to posterior end of the body. Penetration gland cells seven or eight pairs or possibly asymmetric (7 + 8), with large nuclei and fine granular contents. Excretory vesicle Y-shaped, arms extending to posterior level of ventral sucker. Lipid droplets numerous, clustered, medium-sized and small, 2–9 (5) in diameter. Flame cell formula not observed. Tegumental spines dense, backward-directed, in concentric rows, with larger spines from anterior body to anterior level of ventral sucker, and smaller spines from the ventral sucker to the posterior end of the body (Fig. 7A). Sensorial bristles and papillae with long cilia clustering around the stylet opening and oral sucker (Fig. 7B). Small dome-shaped papillae present on and alongside the ventral sucker (Fig. 7C). Distinct patterns of papillae with long sensilla observed on the dorsolateral posterior body (Fig. 7D). No spines or papillae present on tail.

### Cephalogonimidae

*Cephalogonimus* sp.

First intermediate host: *Ampullaceana balthica* (Gastropoda, Lymnaeidae).

Location: Rotbach and Emscher, Germany.

Prevalence: 38.5% (5 of 13 snails) and 3.5% (21 of 595 snails).

Representative DNA sequences: PV248747–752 (28S); PV252992–994 (ITS1-5.8S-ITS2); PV248795–800 (ITS2); PV605631–635; PV605637 and PV605655–658 (*cox*1); and PV602040–041 (*nad*1).

**Fig. 8:**
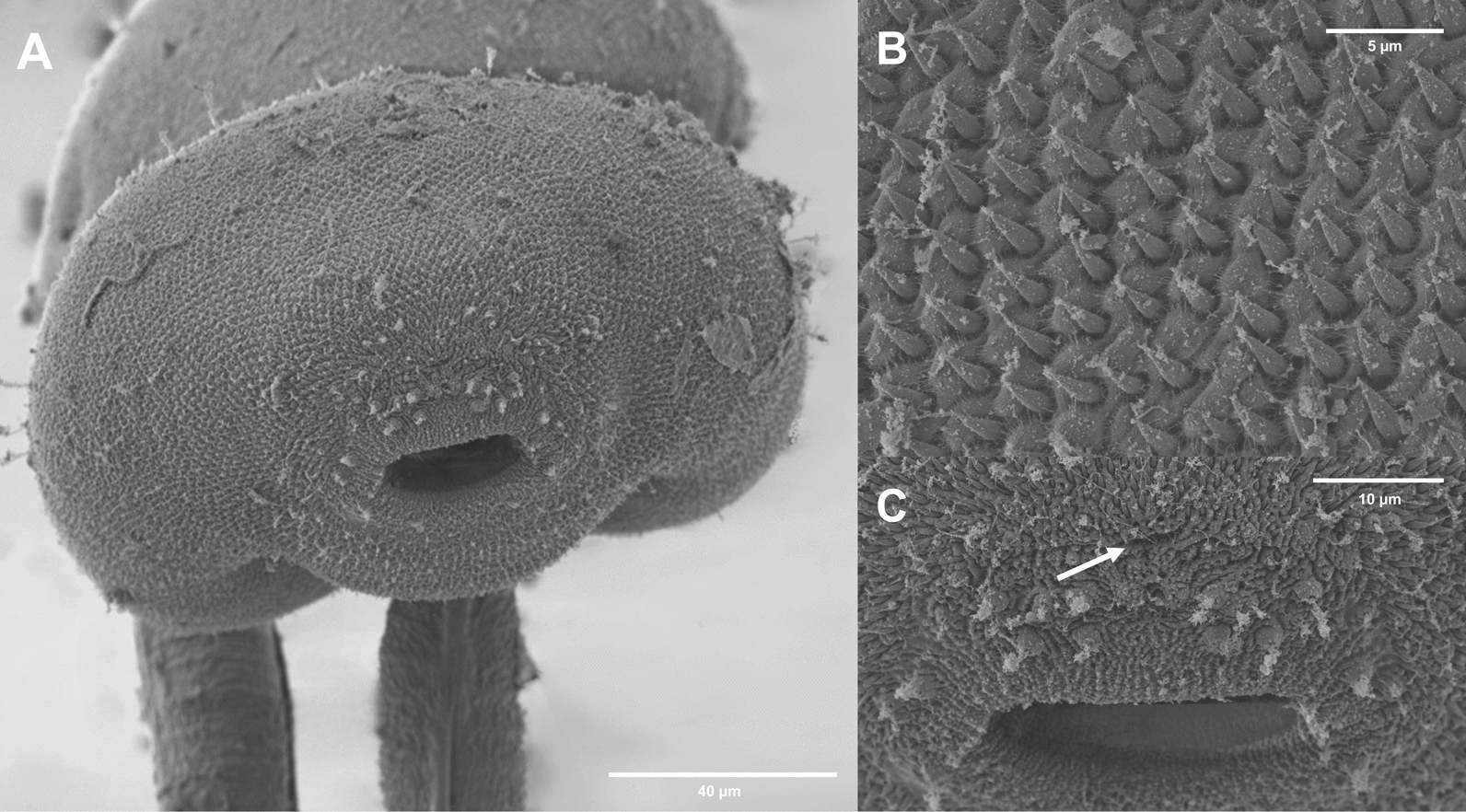
Surface morphology of *Cephalogonimus* sp. (SEM). **A** cercarial body, enface view; **B** dense spines on anterior body; **C** stylet opening (white arrow) and oral sucker with papillae

Cercariae (Figs. 5C, D; 6C, D; 8):

Descriptions and measurements based on ten live specimens and SEM analyses are shown below.

Body elongate oval, maximum width just anterior to ventral sucker; 347–453 $\times$ 186–246 (408 $\times$ 233). Tail simple, 240–301 (274) long, 58–82% (68%) of body length, 63–72 (67) wide at base. Tail with small, continuous fin fold. Oral sucker well-developed, muscular, subspherical, 87–97 $\times$ 96–106 (92 $\times$ 102). Stylet dorsal to mouth opening; small, robust, sharply pointed, 20–23 (22) long. Incomplete basis, slightly thickened; 7–8 (7) wide at base and 5–7 (6) wide above base. Anterior lateral thickening not much pronounced, similar width as base, 7–8 (7). Stylet width at anterior thickening 30–36% (34%) of stylet length. Ventral sucker well-developed, subspherical, equatorial, 64–83 $\times$ 83–91 (77 $\times$ 86). Ventral sucker smaller than oral sucker, width ratio 1:0.81–0.89 (1:0.84). Prepharynx short, pharynx weakly muscular, elongate oval, 28–37 $\times$ 28–34 (34 $\times$ 31). Oesophagus short, muscular, intestinal bifurcation close to ventral sucker. Caeca well-pronounced, reaching to anterior level of excretory vesicle. Excretory vesicle Y-shaped, arms extending to posterior level of ventral sucker. Penetration gland cells numerous, indistinct, with large nuclei, with fine granular contents. Flame cell formula not observed. Fine tegumental spines covering the whole body, dense, backward-directed, in concentric rows. Larger spines on the anterior body, smaller spines from the level of the ventral sucker to the posterior end of the body (Fig. 8A, B). Sensorial bristles and papillae with long cilia cluster around the stylet opening and oral sucker (Fig. 8C). No spines or papillae observed on tail.

### Telorchiidae

#### Opisthioglyphe ranae

First intermediate hosts: *Ampullaceana balthica* and *Stagnicola palustris* (Gastropoda, Lymnaeidae).

Location: Emscher, Germany.

Prevalence: 0.7% (4 of 595 snails) and 2.6% (1 of 39 snails).

Representative DNA sequences: PV248753–756 (28S); PV252995–998 (ITS1-5.8S-ITS2); PV248801–804 (ITS2); and PV605636 and PV605639–642 (*cox*1).

**Fig. 9:**
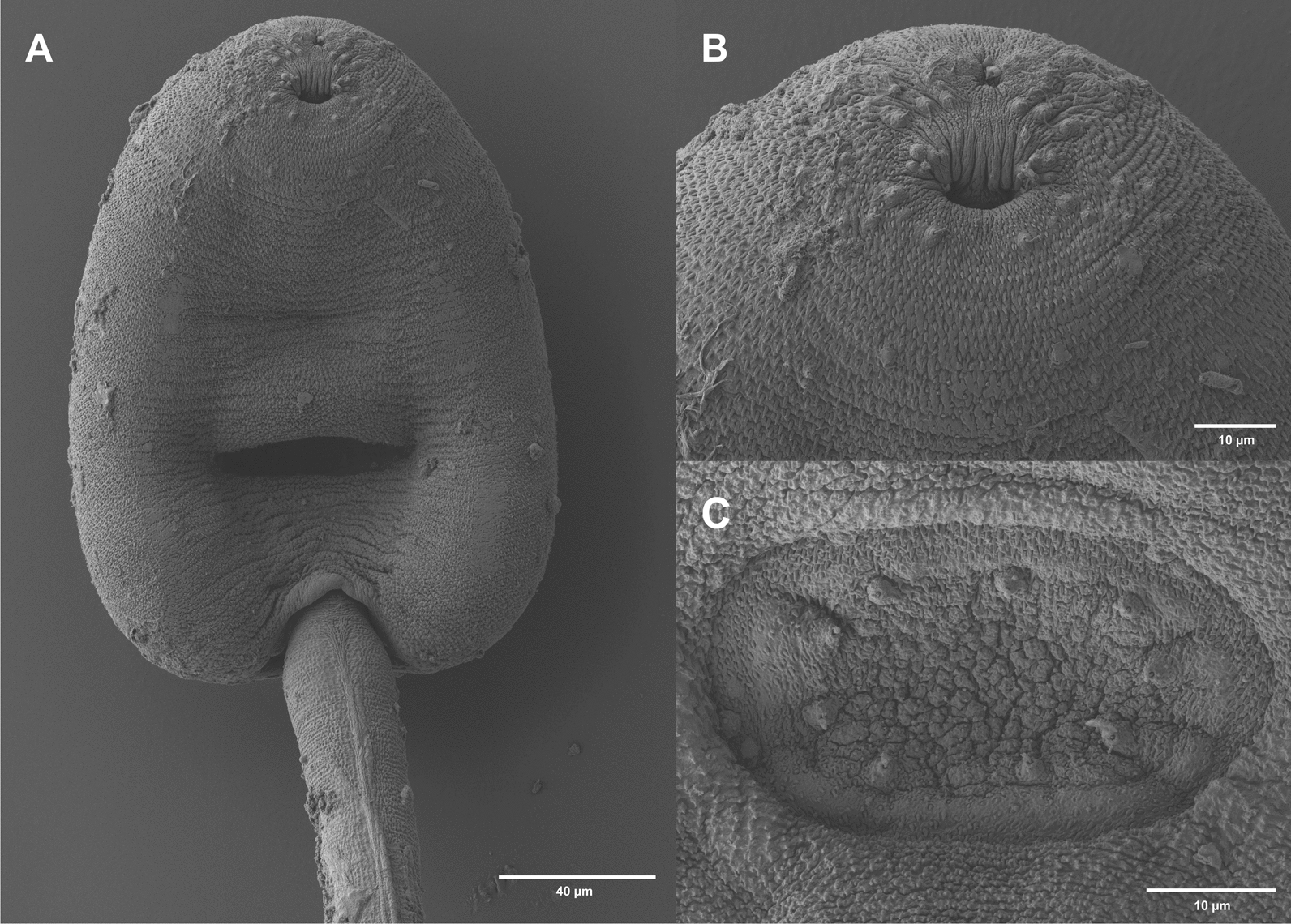
Surface morphology of *O. ranae* (SEM). **A** cercarial body, ventral view; **B** stylet opening and oral sucker with sensorial bristles and papillae; **C** ventral sucker with small dome-shaped papillae

Cercariae (Figs. 5E, F; 6E, F; 9):

Descriptions and measurements based on three live specimens and SEM analyses are shown below.

Body elongate oval, maximum width anterior to ventral sucker; 416–471 $\times$ 222–236 (440 $\times$ 228). Tail simple, 222–234 (229) long, 50–53% (52%) of body length, 56–61 (59) wide at base. Tail with small, continuous fin fold. Oral sucker well-developed, muscular, rounded, 86–90 $\times$ 87–91 (88 $\times$ 89). Stylet dorsal to mouth opening; large, robust, sharply pointed, 28–30 (29) long. Incomplete basis with thickening, 6–7 (6) wide at base and 4–5 (4) wide above base. Anterior lateral thickening not much pronounced, similar width as base, (6). Stylet width at anterior thickening 20–21% (21%) of stylet length. Ventral sucker subspherical, post-equatorial, 85–87 $\times$ 85–92 (87 $\times$ 89). Suckers of similar size, width ratio 1:0.97–1.06 (1:1). Prepharynx short, pharynx feebly muscular, rounded to oval, 28–31 $\times$ 31–34 (29 $\times$ 32). Oesophagus narrow, intestinal bifurcation close to ventral sucker. Caeca reaching to posterior end of the body. Penetration gland cells eight pairs, with large nuclei and fine granular contents. Excretory vesicle Y-shaped, arms extending to posterior level of ventral sucker. Lipid droplets numerous, clustered, small, 2–5 (4) in diameter. Flame cell formula not observed. Tegumental spines covering the whole body, dense, backward-directed, in concentric rows. Larger spines on the anterior body, and smaller spines from the level of the ventral sucker to the posterior body end (Fig. 9A). Sensorial bristles and papillae with long cilia cluster around the stylet opening and oral sucker (Fig. 9B). Small dome-shaped papillae present on the ventral sucker (Fig. 9C). No spines or papillae observed on tail.

### Occurrence and prevalence

The prevalence of the identified amphibian trematode taxa varied considerably between sampling locations and seasons. *Lecithopyge* sp. was exclusively found in the Boye catchment, *Cephalogonimus* sp. occurred at both Emscher and Rotbach sampling sites, and *O. ranae* was only found in the Emscher catchment. A strong seasonal pattern was observed in the prevalence of infections with *Lecithopyge* sp., which was detected exclusively in monthly samplings between December 2023 and June 2024, and again in January 2025. *Cephalogonimus* sp. was found in spring, summer, and autumn, and *O. ranae* was found in summer and autumn samples in 2024 (Tables 3, 4).

**Table 3:** Prevalence data for the identified amphibian trematode taxa from the Boye river

Year	Month/season	Host species	Sample size	Prevalence patent/prepatent [%]					
				*Lecithopyge* sp.		*Cephalogonimus* sp.		*O. ranae*	
Patent data only									
2023	May–November		1459	0	–	0	–	0	–
	December	*A. balthica*	126	3.9	–	0	–	0	–
2024	January	*A. balthica*	153	5.2	–	0	–	0	–
	February	*A. balthica*	153	7.2	–	0	–	0	–
	March	*A. balthica*	208	5.3	–	0	–	0	–
	April	*A. balthica*	193	4.1	–	0	–	0	–
	May	*A. balthica*	152	3.0	–	0	–	0	–
	June	*A. balthica*	143	2.0	–	0	–	0	–
	July		253	0	–	0	–	0	–
Patent and prepatent data									
2024	August–December		1115	0	0	0	0	0	0
2025	January	*A. balthica*	148	0.7	0	0	0	0	0

“–” indicates samples with patent data only

**Table 4:** Prevalence data for the identified amphibian trematode taxa from the Rotbach river and Emscher river

Site and year	Month/season	Host species	Sample size	Prevalence patent/prepatent [%]					
				*Lecithopyge* sp.		*Cephalogonimus* sp.		*O. ranae*	
Patent and prepatent data									
2024									
Rotbach	Spring	*A. balthica*	13	0	0	30.1	7.7	0	0
Emscher	Spring	*A. balthica*	97	0	0	1.0	0	0	0
	Summer	*A. balthica*	154	0	0	1.9	3.9	0	0
		*S. palustris*	25	0	0	0	0	0	4.0
	Autumn	*A. balthica*	342	0	0	3.2	0	0.3	0.9

## Discussion

Investigating aquatic snails as first intermediate hosts of amphibian trematodes enabled us to identify three taxa infecting amphibian populations across Europe. Existing morphological and molecular data on these taxa, especially on morphology of their larval stages, were limited. This study provides a comprehensive dataset combining light and scanning electron microscopy analyses with sequences from multiple genetic markers and insights into seasonal transmission dynamics applicable for future species identification.

In the present study, species-level identification of *Lecithopyge* sp. was constrained by the limited availability of morphological and molecular reference data, which currently includes only a single sequence of *L. rastellus* from Ukraine [45]. Referred to as *Dolichosaccus rastellus* and *Opisthioglyphe rastellus*, this species has been reported from a broad range of amphibian definitive hosts across the families Alytidae, Bombinatoridae, Bufonidae, Ranidae, and Salamandridae from Europe, Russia, and the Middle East (Additional File 4: Supplementary Table S9). Its life cycle involves lymnaeid snails as the first intermediate hosts [46, 47] and mayfly larvae as second intermediate hosts [48], while experimental life cycle reconstructions have also successfully included *Chironomus* spp. and other arthropod larvae [46, 49]. However, Grabda-Kazubska [29] indicated an abbreviated life cycle as the most common infection route in which a single amphibian specimen functions as both the second intermediate and the definitive host. Such obligate or facultative life cycle abbreviations are present in several amphibian trematode species and appear to be common key adaptations that enhance trematode persistence and life cycle completion in amphibian hosts [50, 51]. On the basis of morphological characteristics, *Lecithopyge* was originally classified as a subgenus of *Dolichosaccus* within the Opisthioglyphinae, a subfamily of the Telorchiidae [52]. Although an initial phylogenetic analysis placed *Lecithopyge* as a separate genus within the Plagiorchiidae [45], which would be supported by our 28S rDNA and ITS1-5.8S-ITS2 phylogenies, recent literature exclusively refers to *L. rastellus* under its synonyms without providing any molecular data (Additional File 4: Supplementary Table S9). The lack of clustering among the sequences of *Lecithopyge* with either *Opisthioglyphe* or *Dolichosaccus* in our phylogenetic analyses highlights this unresolved taxonomy and the need for a more comprehensive molecular investigation to clarify their phylogenetic relationships. In our samplings, *Lecithopyge* sp. was documented solely in *A. balthica*, indicating host specificity for the first intermediate host. While the focus on patent infections in the Boye catchment limits the scope of interpretation, the transmission from the first intermediate host (cercarial shedding) appears to follow strong seasonal patterns. This seasonality likely corresponds with the reproductive season of several amphibian species in Europe and would therefore facilitate infection of amphibian larvae as the second intermediate host. Accordingly, the timing of definitive amphibian hosts returning to the spawning sites, either in autumn to hibernate in the water or in early spring for mating and reproduction, could mark the introduction of trematode eggs into the water body and the subsequent infection of first intermediate snail hosts [53, 54].

The species identified as *Cephalogonimus* sp. in our study differed from available records of *C. retusus* and *C. americanus*. Within the genus *Cephalogonimus*, controversy exists about the differentiation between *C. retusus* and *C. europaeus*; while some authors consider these two as synonymous [55], others address them as distinct species [56]. As no molecular or larval morphological data were available for *C. europaeus*, we were not able to compare our identified lineage to representatives of this species and thus cannot exclude them to be conspecifics. So far, records of the life cycles of Eurasian *Cephalogonimus* spp. are limited to *C. retusus* and *C. europaeus*, with definitive hosts including members of the Ranidae and Pelobatidae as well as reptile species of families Colubridae and Psammophiidae (Additional File 4: Supplementary Table S9). Lymnaeid snails of the genera *Galba* and *Lymnaea* have been reported as first intermediate hosts in the life cycle of *C. europaeus* and undefined *Cephalogonimus* spp. [56, 57]. Metacercariae of *Cephalogonimus* spp. have been documented from tadpoles or newt larvae, which are then either ingested by adult amphibians or mature into adult trematodes following metamorphosis of the host [58, 59]. In our study, we observed active shedding of *Cephalogonimus* sp. cercariae in spring, summer, and autumn months, with the highest prevalence of patent infections in autumn. As transmission patterns of trematode cercariae are commonly synchronized with second intermediate host availability, these shedding patterns could hint at the availability of suitable second intermediate hosts other than amphibian larvae, which would be available during summer and autumn months [60, 61]. A comprehensive revision of the morphological characteristics of *C. retusus* and *C. europaeus*, along with the generation of molecular data for these and other species within the genus, will be needed to resolve their taxonomy and help clarify the position within the Cephalogonimidae for the lineage identified here.

*Opisthioglyphe ranae* is one of the most common amphibian trematode species across Europe and has been reported from a broad host range, spanning the families Bombinatoridae, Bufonidae, Hylidae, and Ranidae, as well as snake species *Natrix natrix* (Colubridae) (Additional File 4: Supplementary Table S9). Despite its widespread distribution and several recent records of different life stages (e.g., [22, 62–65]), studies linking morphological and molecular data for species identification remain scarce [22, 62]. The life cycle of *O. ranae* involves lymnaeid snails as the first intermediate and also as potential second intermediate hosts [31, 66]. Metacercariae have also been recorded in bivalves and a fish species [62, 67]; however, an abbreviated life cycle in which amphibians serve as both second intermediate and definitive hosts is again assumed to be the preferred route of infection [29]. In our dataset, infections with *O. ranae* were infrequent and limited to summer and autumn months, including a single patent infection in autumn. These observations are consistent with the species’ capacity to utilize a broader range of second intermediate hosts, which may reduce its dependence on seasonal or successional transmission patterns.

Several echinostome trematodes are recognized for infecting tadpoles as second intermediate hosts, often with significant effects on tadpole survival, growth, and development [68]. In our study, we documented *E. recurvatum*, a cosmopolitan echinostome with broad distribution and a wide range of freshwater snails as first intermediate hosts. Accordingly, we observed infections in four snail species from the families Lymnaeidae and Planorbidae. Typically, molluscs are reported as second intermediate hosts and a variety of birds as definitive hosts in the life cycle [69]. Two studies have also documented loaches and tadpoles as possible second intermediate hosts in experimental infections [70, 71], and *E. recurvatum* has recently been recorded from amphibians in Latvia [65]. However, these records relied exclusively on morphological identification, despite evidence that echinostomes are often cryptic and difficult to distinguish without molecular tools [72, 73]. This lack of molecular species delimitation together with the complexity of *Echinoparyphium* spp. life cycles complicates accurate determination of transmission routes [69]. As a result, the role of *E. recurvatum* in amphibian infections remains unclear.

The current study was constrained by the limited availability of relevant reference sequences for several trematode families in GenBank, which hinders accurate species delimitation and robust phylogenetic inference. In addition, the life cycles of many trematode species remain unresolved, impeding the ability to accurately identify all amphibian-infecting trematode species and link developmental stages across hosts. Representatives of the genera identified in our dataset have been documented from a broad spectrum of amphibian definitive hosts, yet trematode species were identified primarily with morphological data (Additional File 4: Supplementary Table S9). The implementation of molecular methods in past years has increasingly revealed that several trematode taxa previously thought to exhibit such broad host ranges actually comprise multiple cryptic species, each with high individual host specificity (e.g., [74–79]). An “integrative” approach to species delimitation will thus be essential to verify or re-evaluate species identity and host specificity of amphibian trematodes in future works [80]. Our study advances this objective by providing detailed information on the taxa identified here.

## Conclusions

Taken together, our findings and the limitations encountered underscore the need for comprehensive research on amphibian trematodes, integrating both morphological and molecular data. We demonstrated that targeting intermediate hosts is an effective approach for investigating trematodes of rare or protected amphibian hosts that cannot be extensively sampled. Generating reliable links between morphological characteristics, molecular data, and life cycle dynamics will refine our understanding of amphibian parasite diversity and facilitate application of future noninvasive approaches such as roadkill parasitological investigations or eDNA approaches.

## Supplementary Information

Additional file 1. Table S1. Summary of all sequences of trematodes used for 28S and ITS phylogenetic analyses. Novel sequences generated in this study are highlighted in bold. Life cycle stage: A adult, C cercaria, MC metacercaria.

Additional file 2. Table S2: Uncorrected p-distances of the 28S rDNA alignment for representatives of the Plagiorchioidea (1,151 nt, primer pair digl2/1500R). Table S3: Uncorrected p-distances of the ITS1-5.8S-ITS2 rDNA alignment for representatives of the Plagiorchioidea (682 nt, primer pair D1/D2). Table S4: Uncorrected p-distances of the ITS2 rDNA alignment (410 nt, primer pair 3S/ITS2). Table S5: Uncorrected p-distances of the *cox*1 alignment (750 nt, primer pair Dice1F/Dice14R). Table S6: Uncorrected p-distances of the *cox*1 alignment (341 nt, primer pair JB3/JB4.5). Table S7: Uncorrected p-distances of the *nad*1 alignment (456 nt, primer pair NDJ11/NDJ2a).

Additional file 3. Table S8: Summary of the recorded trematode species in all collected gastropod species and their second intermediate and definitive host groups.

Additional file 4. Table S9: Overview of species records of *Lecithopyge *sp., *Cephalogonimus *sp., and *Opisthioglyphe ranae* in amphibians (and reptiles) from published literature. Life stage: A adult, J juvenile, MC metacercaria. The records included in this table were compiled through a literature search in Scopus and Web of Science using combinations of the search terms: (Lecithopyge OR Dolichosaccus OR Cephalogonimus OR Opisthioglyphe OR "Opisthioglyphe ranae" OR rastellus) AND (amphib* OR frog* OR salamander* OR newt* OR toad*). Publications were screened for further relevant citations. Both abstracts and full texts were reviewed to extract occurrence data.

## Data Availability

Sequence data generated for this study have been deposited in GenBank (accession nos. PV248741–PV248756, PV248788–PV248804, PV252985–PV252995, PV602038–PV602041, PV605625–PV605642, and PV605649–PV605658). The raw data that support the findings of this study are available from the corresponding author upon reasonable request.
