# Supplementary material for: Intermediate insights: tracing trematodes infecting amphibians via their first intermediate snail hosts
Source: Parasit Vectors. 2025 Jul 15;18:285. doi: 10.1186/s13071-025-06920-x (PMC12265291; doi:10.1186/s13071-025-06920-x)
Supplement: Supplementary file 1 — Additional file 1. Table S1. Summary of all sequences of trematodes used for 28S and ITS phylogenetic analyses. Novel sequences generated in this study are highlighted in bold. Life cycle stage: A adult, C cercaria, MC metacercaria. [file 13071_2025_6920_MOESM1_ESM.docx]

Additional File 1: Table S1: Summary of all sequences of trematodes used for 28S and ITS phylogenetic analyses. Novel sequences generated in this study are highlighted in bold. Life cycle stage: A adult, C cercaria, MC metacercaria.

| Taxon | Life cycle stage | Host | Country | GenBank Accession No. | | Reference |
| --- | --- | --- | --- | --- | --- | --- |
|  |  |  |  | 28S | ITS1-5.8S-ITS2 |  |
| Superfamily Plagiorchioidea, Lühe 1901 | | | | | | |
| Family Auridistomidae | | | | | | |
| *Auridistomum chelydrae* | A | *Chelydra serpentine* | USA | AY116872 | – | [1] |
| Family Brachycoeliidae | | | | | | |
| *Brachycoelium salamandrae* | A | *Salamandra salamandra* | Ukraine | AF151935 | – | [2] |
| Family Cephalogonimidae | | | | | | |
| *Cephalogonimus americanus* | A | *Ambystoma velasci* | Mexico | HM137615 | – | [3] |
| *Cephalogonimus retusus* | A | *Pelophylax ridibundus* | Bulgaria | AY222276 | – | [1] |
| ***Cephalogonimus* sp.** | **C** | ***Ampullaceana balthica*** | **Germany** | **PV248747-52** | **PV252992-94** | **This study** |
| Family Choanocotylidae | | | | | | |
| *Choanocotyle hobbsi* | A | *Chelodina oblonga* | Australia | EU196356 | | [4] |
| *Choanocotyle nematoides* | A | *Emydura kreftii* | Australia | EU196358 | – | [4] |
| *Choanocotyle platti* | A | *Chelodina rugosa* | Australia | – | EU196355 | [4] |
| Family Dolichoperoididae | | | | | | |
| *Dolichoperoides macalpini* | A | *Notechis scutatus* | Australia | OK572363 | – | [5] |
|  | A | *Notechis scutatus* | Australia | – | OM568839 | [5] |
| Family Glypthelminthidae | | | | | | |
| *Glypthelmins facioi* | A | *Rana* sp. | Costa Rica | AY875675 | – | [6] |
| *Glypthelmins pennsylvaniensis* | A | *Pseudacris crucifer crucifer* | USA | HM137608 | – | [3] |
| *Glypthelmins quieta* | A | *Rana catesbeiana* | USA | AY222278 | – | [1] |
| Family Haematoloechidae | | | | | | |
| *Hameatoloechus asper* | A | *Rana arvalis* | Ukraine | AF151934 | – | [2] |
| *Haematoloechus breviplexus* | A | *Rana catesbeiana* | USA | – | AF387796 | [7] |
| *Haematoloechus complexus* | A | *Rana blairi* | USA | – | AF316155 | [7] |
| *Haematoloechus longiplexus* | A | *Rana catesbeiana* | USA | AY222280 | – | [1] |
| *Haematoloechus variegatus* | A | *Rana arvalis* | Ukraine | AF151916 | – | [8] |
| Family Leptophallidae | | | | | | |
| *Leptophallus nigrovenosus* | A | *Natrix natrix* | Ukraine | AF151914 | – | [8] |
| Family Macroderoididae | | | | | | |
| *Macroderoides texanus* | A | *Atractosteus spatula* | USA | EU850398 | | [9] |
| *Macroderoides trilobatus* | A | *Lepisosteus platyrhincus* | USA | – | EU850406 | [9] |
| *Perezitrema bychowskyi* | A | *Atractosteus tropicus* | Mexico | KU535686 | – | [10] |
| Family Mesocoeliidae | | | | | | |
| *Mesocoelium brevicaecum* | A | *Bufo japonicus formosus* | Japan | LC770434 | – | [11] |
| *Mesocoelium japonicum* | A | *Pareas atayal* | Japan | LC770436 | – | [11] |
| Family Omphalometridae | | | | | | |
| *Omphalometra flexuosa* | C | *Planorbis planorbis* | Poland | AF300333 | – | [12] |
| Family Orientocreadiidae | | | | | | |
| *Orientocreadium batrachoides* | A | *Clarias gariepinus* | Mozambique | MK496882 | – | [13] |
| *Orientocreadium pseudobagri* | A | *Perccottus glenii* | Russia | MF611697 | – | [14] |
| Family Plagiorchiidae | | | | | | |
| *Aptorchis aequalis* | A | *Emydura krefftii* | Australia | EF014729 | – | [15] |
|  | A | *Emydura krefftii* | Australia | – | EU334369 | [16] |
| *Aptorchis megacetabulus* | A | *Chelodina rugosa* | Australia | – | EF014730 | [15] |
| *Aptorchis megapharynx* | A | *Chelodina longicollis* | Australia | EF014727 | – | [15] |
| *Lecithopyge rastellus* | A | *Bombina variegata* | Ukraine | AF151932 | – | [2] |
| ***Lecithopyge* sp.** | **C** | ***Ampullaceana balthica*** | **Germany** | **PV248741-46** | **PV252985-91** | **This study** |
| *Haplometra cylindracea* | A | *Rana arvalis* | Ukraine | AF151933 | – | [2] |
| *Plagiorchis elegans* | A | *Apodemus sylvaticus* | UK | – | JX522536 | [17] |
| *Plagiorchis koreanus* | A | *Pipistrellus pipistrellus* | England | – | JF784194 | [18] |
| *Plagiorchis maculosus* | A | *Fringilla coelebs* | Ukraine | – | AF316152 | [7] |
| *Plagiorchis muelleri* | C | *Ampullaceana balthica* | Ireland | MW528603 | – | [19] |
|  | A | *Eptesicus serotinus* | Ukraine | – | AF151947 | [20] |
| *Plagiorchis vespertilionis* | MC | *Myotis daubentoni* | Ukraine | AF151931 | – | [2] |
|  | A | *Myotis daubentoni* | Ukraine | – | AF151949 | [20] |
| Family Reniferidae | | | | | | |
| *Renifer aniarum* | A | *Nerodia rhombifer* | USA | HQ665459 | – | [21] |
| *Renifer kansensis* | A | *Elaphe quadrivirgata* | Japan | LC823737 | – | [22] |
| Family Telorchiidae | | | | | | |
| *Dolichosaccus symmetrus* | A | – | Australia | – | L01631 | [23] |
| *Dolichosaccus* sp. | A | – | Australia | – | L01630 | [23] |
| *Opisthioglyphe ranae* | A | *Rana arvalis* | Ukraine | AF151929 | – | [2] |
|  | **C** | ***Stagnicola palustris*** | **Germany** | **PV248753** | **PV252995** | **This study** |
|  | **C** | ***Ampullaceana balthica*** | **Germany** | **PV248754-56** | **PV252996-98** | **This study** |
| *Telorchis assula* | A | *Natrix natrix* | Ukraine | AF151915 | – | [8] |
| *Telorchis bonnerensis* | A | *Lithobates sylvaticus* | USA | – | JF820593 | [24] |
| *Telorchis corti* | C | *Radix coreana* | South Korea | ON792562 | – | [25] |
| Superfamily Monorchioidea Odhner, 1911 | | | | | | |
| Family Lissorchiidae | | | | | | |
| *Lissorchis kritskyi* | A | *Carpiodes cyprinus* | USA | AY222250 | – | [1] |
|  | A | *Carpiodes velifer* | Vietnam | – | MT928329 | [26] |

**References**

1. Olson PD, Cribb TH, Tkach VV, Bray RA, Littlewood DTJ. Phylogeny and classification of the Digenea (Platyhelminthes: Trematoda). Int J Parasitol. 2003; doi:10.1016/s0020-7519(03)00049-3.

2. Tkach V, Pawlowski J, Mariaux J. Phylogenetic analysis of the suborder plagiorchiata (Platyhelminthes, Digenea) based on partial lsrDNA sequences. Int J Parasitol. 2000; doi:10.1016/s0020-7519(99)00163-0.

3. Razo-Mendivil U, de León GP-P. Testing the evolutionary and biogeographical history of *Glypthelmins* (Digenea: Plagiorchiida), a parasite of anurans, through a simultaneous analysis of molecular and morphological data. Mol Phylogenet Evol. 2011; doi:10.1016/j.ympev.2011.02.018.

4. Tkach V, Snyder S. *Choanocotyle platti* sp. nov. from the northern long-necked turtle, *Chelodina rugosa* (Pleurodira, Chelidae) in Australia. Acta Parasitol 2007. doi:10.2478/s11686-007-0057-5.

5. Barton DP, Lettoof DC, Fearn S, Zhu X, Francis N, Shamsi S. *Dolichoperoides macalpini* (Nicoll, 1914) (Digenea: Dolichoperoididae) infecting venomous snakes (Elapidae) across Australia: molecular characterisation and infection parameters. Parasitol Res. 2022; doi:10.1007/s00436-022-07502-x.

6. Razo-Mendivil UJ, León-Règagnon V, Pérez-Ponce de León G. Description of two new species of *Glypthelmins* Stafford, 1905 (Digenea: Macroderoididae) in *Rana* spp. from Mexico, based on morphology and mtDNA and rDNA sequences. Syst Parasitol. 2004; doi:10.1023/B:SYPA.0000048099.73779.f4.

7. Snyder SD, Tkach VV. Phylogenetic and biogeographical relationships among some Holarctic frog lung flukes (Digenea: Haematoloechidae). J Parasitol. 2001; doi:10.1645/0022-3395(2001)087[1433:PABRAS]2.0.CO;2.

8. Tkach VV, Grabda-Kazubska B, Pawlowski J, Swiderski S. Molecular and morphological evidence for close phylogenetic affinities of the genera *Macrodera*, *Leptophallus*, *Metaleptophallus* and *Paralepoderma* (Digenea, Plagiorchiata). Acta Parasitol. 1999:170–9.

9. Tkach VV, Strand EJ, Froese L. *Macroderoides texanus* n. sp. (Digenea: Macroderoididae) from alligator gar, *Atractosteus spatula* in Texas. Parasitol Res. 2008; doi:10.1007/s00436-008-1155-5.

10. Hernández-Mena DI, Mendoza-Garfias B, Ornelas-García CP, Pérez-Ponce de León G. Phylogenetic position of *Magnivitellinum* Kloss, 1966 and *Perezitrema* Baruš & Moravec, 1967 (Trematoda: Plagiorchioidea: Macroderoididae) inferred from partial 28S rDNA sequences, with the establishment of Alloglossidiidae n. fam. Syst Parasitol. 2016; doi:10.1007/s11230-016-9645-9.

11. Tsuchida K, Urabe M, Nishikawa K, Hoso M, Wu S. Addressing the taxonomic confusion of *Mesocoelium* Odhner, 1910 (Trematoda: Plagiorchioidea: Mesocoeliidae) in Japanese urodelan and anuran amphibians. Syst Parasitol. 2024; doi:10.1007/s11230-023-10126-z.

12. Tkach V, Grabda-Kazubska B, Swiderski Z. Systematic position and phylogenetic relationships of the family Omphalometridae (Digenea, Plagiorchiida) inferred from partial lsrDNA sequences. Int J Parasitol. 2001; doi:10.1016/s0020-7519(00)00154-5.

13. Dumbo JC, Dos Santos QM, Avenant-Oldewage A. morphological and molecular characterization of *Glossidium pedatum* Looss, 1899 and *Orientocreadium batrachoides* Tubangui, 1931 from sharptooth catfish*, Clarias gariepinus* (Burchell, 1822). Afr Zool. 2019; doi:10.1080/15627020.2019.1595143.

14. Sokolov SG, Shchenkov SV. Phylogenetic position of the family Orientocreadiidae within the superfamily Plagiorchioidea (Trematoda) based on partial 28S rDNA sequence. Parasitol Res. 2017; doi:10.1007/s00436-017-5594-8.

15. Tkach VV, Snyder SD. *Aptorchis megacetabulus* n. sp. (Platyhelminthes: Digenea) from the northern long-necked turtle, *Chelodina rugosa* (Pleurodira: Chelidae), in Australia. J Parasitol. 2007; doi:10.1645/GE-998R.1.

16. Tkach VV, Snyder SD. *Aptorchis glandularis* n. sp. (Digenea: Plagiorchioidea) from the northwestern red-faced turtle*, Emydura australis,* (Pleurodira: Chelidae) in the Kimberley, Western Australia. J Parasitol. 2008; doi:10.1645/GE-1439.1.

17. Boyce K, Hide G, Craig PS, Reynolds C, Hussain M, Bodell AJ, et al. A molecular and ecological analysis of the trematode *Plagiorchis elegans* in the wood mouse *Apodemus sylvaticus* from a periaquatic ecosystem in the UK. J Helminthol. 2014; doi:10.1017/S0022149X13000199.

18. Lord JS, Parker S, Parker F, Brooks DR. Gastrointestinal helminths of pipistrelle bats (*Pipistrellus pipistrellus/Pipistrellus pygmaeus*) (Chiroptera: Vespertilionidae) of England. Parasitology. 2012; doi:10.1017/S0031182011002046.

19. Kudlai O, Pantoja C, O’Dwyer K, Jouet D, Skírnisson K, Faltýnková A. Diversity of *Plagiorchis* (Trematoda: Digenea) in high latitudes: Species composition and snail host spectrum revealed by integrative taxonomy. J. Zool. Syst. Evol. Res. 2021; doi:10.1111/jzs.12469.

20. Tkach VV, Pawlowski J, Sharpilo VP. Molecular and morphological differentiation between species of the *Plagiorchis vespertilionis* group (Digenea, Plagiorchiidae) occurring in European bats, with a re-description of *P. vespertilionis* (Müller, 1780). Syst Parasitol. 2000; doi:10.1023/a:1006358524045.

21. Santoro M, Tkach VV, Mattiucci S, Kinsella JM, Nascetti G. *Renifer aniarum* (Digenea: Reniferidae), an introduced North American parasite in grass snakes *Natrix natrix* in Calabria, southern Italy. Dis Aquat Organ. 2011; doi:10.3354/dao02365.

22. Waki T, Yasuda M, Seo H, Tokiwa T. A record of artificial transportation of a snake infected with introduced trematodes to an area where infection was unreported. Jpn. J. Zoo Wildl. Med. 2024; doi:10.5686/jjzwm.29.81.

23. Luton K, Walker D, Blair D. Comparisons of ribosomal internal transcribed spacers from two congeneric species of flukes (Platyhelminthes: Trematoda: Digenea). Mol Biochem Parasitol. 1992; doi:10.1016/0166-6851(92)90181-i.

24. Pulis EE, Tkach VV, Newman RA. Helminth parasites of the Wood Frog, *Lithobates sylvaticus*, in Prairie Pothole Wetlands of the Northern Great Plains. Wetlands. 2011; doi:10.1007/s13157-011-0183-6.

25. Aksenova OV. Intermediate hosts of trematodes of the family Telorchiidae Looss, 1899. 2024; Unpublished.

26. Truong TN, Warren MB, Ksepka SP, Curran SS, Bullard SA. *Posthovitellinum psiloterminae* n. gen., n. sp. (Digenea: Lissorchiidae) infecting the intestine of *Cyclocheilos enoplos* (Cypriniformes: Cyprinidae) in the Mekong River, Vietnam. J Parasitol. 2021; doi:10.1645/20-77.
