# Supplementary material for: Intermediate insights: tracing trematodes infecting amphibians via their first intermediate snail hosts
Source: Parasit Vectors. 2025 Jul 15;18:285. doi: 10.1186/s13071-025-06920-x (PMC12265291; doi:10.1186/s13071-025-06920-x)
Supplement: Supplementary file 3 — Additional file 3. Table S8: Summary of the recorded trematode species in all collected gastropod species and their second intermediate and definitive host groups. [file 13071_2025_6920_MOESM3_ESM.docx]

Additional File 3: Table S8: Summary of the recorded trematode species in all collected gastropod species and their second intermediate and definitive host groups.

| Trematode family  and species | First intermediate host (this study) | Second intermediate host (literature) | | Definitive host group (literature) | | Reference | |
| --- | --- | --- | --- | --- | --- | --- | --- |
| **Cephalogonimidae** | | | | | | |  |
| *Cephalogonimus* sp. | *A. balthica* | Amphibians | Amphibians | | [1, 2] | |  |
| **Echinostomatidae** | | | | | | |  |
| *Echinoparyphium recurvatum* | *A. balthica,  L. stagnalis,  S. palustris, P. carinatus* | Molluscs, (tadpoles, fish) | Anatidae | | [3-5] | |  |
| *Hypoderaeum conoideum* | *A. balthica,  L. stagnalis* | Molluscs | Waterfowl | | [3] | |  |
| *Moliniella anceps* | *S. palustris* | Molluscs | Rallidae | | [3] | |  |
| *Petasiger phalacrocoracis* | *A. balthica* | Fishes | Cormorants | | [6] | |  |
| **Lecithodendriidae** | | | | | | |  |
| *Lecithodendrium linstowi* | *B. tentaculata* | Unknown (Insect larvae) | Bats | | [7] | |  |
| **Notocotylidae** | | | | | | |  |
| *Notocotylus* sp. (not further identified) | *A. balthica,  S. palustris* | None (encyst on vegetation/substrate) | Anatidae | | [3] | |  |
| *Notocotylus* sp. AK-2017 | *A. balthica,  S. palustris* | None (encyst on vegetation/substrate) | Anatidae | | [3,8] | |  |
| **Plagiorchiidae** | | | | | | |  |
| *Lecithopyge* sp. | *A. balthica* | Insect larvae, amphibians | Amphibians | | [9] | |  |
| *Plagiorchis* sp. (not further identified) | *A. balthica, L. stagnalis,  S. palustris* | – | – | | – | |  |
| *Plagiorchis* sp. 2 | *A. balthica* | Mollusks, Crustaceans | Unknown | | [8] | |  |
| *Plagiorchis* sp. 3 | *A. balthica* | Insect larvae | Unknown | | [8] | |  |
| *Plagiorchis* sp. 7 | *A. balthica* | Unknown | Unknown | | [8] | |  |
| *P. elegans* | *L. stagnalis,  S. palustris* | Insect larvae, amphipods, mollusks | Birds, mammals, reptiles | | [3,10-11] | |  |
| *P. koreanus* | *A. balthica* | Insect larvae | Bats | | [10] | |  |
| *P. muelleri* | *A. balthica* | Insect larvae | Bats | | [10] | |  |
| *P. vespertilionis* | *A. balthica* | Insect larvae | Bats | | [10] | |  |
| **Pleurogenidae** | | | | | | |  |
| *Leyogonimus polyoon* | *B. tentaculata* | Unknown | Rallidae | | [12] | |  |
| Pleurogenidae gen. sp. 2 | *B. tentaculata* | Unknown | Rallidae | | [12] | |  |
| **Prosthogonimidae** |  |  |  | |  | |  |
| *Prosthogonimus ovatus* | *B. tentaculata* | Insect larvae | Birds | | [13] | |  |
| **Schistosomatidae** | | | | | | |  |
| *Trichobilharzia* sp. (not further identified) | *A. balthica* | None (direct infection) | Anatidae | | [3] | |  |
| **Strigeidae** | | | | | | |  |
| *Apatemon gracilis* | *A. balthica* | Fishes | Fish-eating birds | | [11] | |  |
| *Australapatemon burti* | *A. balthica,  L. stagnalis,  P. carinatus* | Leeches | Anatidae | | [3] | |  |
| *Cotylurus* sp. (not further identified) | *A. balthica* | – | – | | – | |  |
| **Telorchiidae** | | | | | | |  |
| *Opisthioglyphe ranae* | *A. balthica,  S. palustris* | Amphibians, tadpoles | Amphibians | | [3,9] | |  |

**References:**

1. Lang BZ. The life cycle of *Cephalogonimus americanus* Stafford, 1902 (Trematoda: Cephalogonimidae). J Parasitol. 1968; doi:10.2307/3277126.

2. Dronen NO, Lang BZ. The life cycle of *Cephalogonimus salamandrus* sp. n. (Digenea: Cephalogonimidae) from *Ambystoma tigrinum* (Green) from Eastern Washington. J Parasitol. 1974; doi:10.2307/3278681.

3. Brown R, Soldánová M, Barrett J, Kostadinova A. Small-scale to large-scale and back: larval trematodes in *Lymnaea* *stagnalis* and *Planorbarius corneus* in Central Europe. Parasitol Res. 2011; doi:10.1007/s00436-010-2047-z.

4. Bespalaya YV, Kondakov AV, Travina OV, Khrebtova IS, Kropotin AV, Aksenova OV, et al. First record of metacercariae trematodes *Opisthioglyphe ranae* (Digenea: Telorchiidae) and *Echinostoma bolschewense* (Digenea: Echinostomatidae) in *Dreissena polymorpha* (Bivalvia: Dreissenidae) from the Don and Volga river basins, Russia. Ecol Monten. 2022; doi:10.37828/em.2022.54.8.

5. Sokolov SG, Protasova EN, Reshetnikov AN. Parasite fauna of rotan *Perccottus glenii* Dybowski, 1877 (Osteichthyes, Odontobutidae) in some waterbodies of European Russia. Biol Bull Russ Acad Sci. 2013; doi:10.1134/S1062359013100087.

6. Našincová V, Scholz T, Moravec F. Redescription of *Petasiger exaeretus* Dietz, 1909 and *P. phalacrocoracis* (Yamaguti, 1939) (Trematoda: Echinostomatidae), parasites of cormorants. Parasitol Res. 1994; doi:10.1007/BF00012272.

7. Enabulele EE, Lawton SP, Walker AJ, Kirk RS. Molecular and morphological characterization of the cercariae of *Lecithodendrium linstowi* (Dollfus, 1931), a trematode of bats, and incrimination of the first intermediate snail host, *Radix balthica*. Parasitol. 2017; doi:10.1017/s0031182017001640.

8. Soldánová M, Georgieva S, Roháčová J, Knudsen R, Kuhn JA, Henriksen EH, Siwertsson A, Shaw JC, Kuris AM, Amundsen PA, Scholz T, Lafferty KD, Kostadinova A. Molecular analyses reveal high species diversity of trematodes in a sub-Arctic lake. Int J Parasitol. 2017; doi:10.1016/j.ijpara.2016.12.008.

9. Grabda-Kazubska B. Studies on abbreviation of the life cycle in *Opisthioglyphe ranae* (Frölich, 1791) and *O. rastellus* (Olsson, 1876) (Trematoda: Plagiorchiidae). Acta Parasitol Pol. 1969:249–69.

10. Kirillova NY, Kirillov AA, Shchenkov SV, Knyazev AE, Vekhnik VA. Morphological and molecular characterization of plagiorchiid trematodes (*Plagiorchis*: Plagiorchiidae, Digenea) from bats with redescription of *Plagiorchis mordovii* Shaldybin, 1958. J Helminthol. 2024; doi:10.1017/S0022149X23000913.

11. Selbach C, Soldánová M, Feld CK, Kostadinova A, Sures B. Hidden parasite diversity in a European freshwater system. Sci Rep. 2020; doi:10.1038/s41598-020-59548-5.

12. Schwelm J, Kudlai OK, Smit NJ, Selbach S, Sures B. High parasite diversity in a neglected host: larval trematodes of *Bithynia tentaculata* in Central Europe. J Helminthol. 2020; doi:10.1017/S0022149X19001093.

13. Tkach VV, Snyder SD, Świderski Z. On the phylogenetic relationship of some members of Macroderoididae and Ochetosomatidae (Digenea, Plagiorchioidea). Acta Parasitol. 2001;46,267–275.
