# Supplementary material for: Intermediate insights: tracing trematodes infecting amphibians via their first intermediate snail hosts
Source: Parasit Vectors. 2025 Jul 15;18:285. doi: 10.1186/s13071-025-06920-x (PMC12265291; doi:10.1186/s13071-025-06920-x)
Supplement: Supplementary file 4 — Additional file 4. Table S9: Overview of species records of Lecithopyge sp., Cephalogonimus sp., and Opisthioglyphe ranae in amphibians (and reptiles) from published literature. Life stage: A adult, J juvenile, MC metacercaria. The records included in this table were compiled through a literature search in Scopus and Web of Science using combinations of the search terms: (Lecithopyge OR Dolichosaccus OR Cephalogonimus OR Opisthioglyphe OR "Opisthioglyphe ranae" OR rastellus) AND (amphib* OR frog* OR salamander* OR newt* OR toad*). Publications were screened for further relevant citations. Both abstracts and full texts were reviewed to extract occurrence data. [file 13071_2025_6920_MOESM4_ESM.docx]

Additional File 4: Table S9: Overview of species records of *Lecithopyge* sp., *Cephalogonimus* sp., and *Opisthioglyphe ranae* in amphibians (and reptiles) from published literature. Life stage: A adult, J juvenile, MC metacercaria. The records included in this table were compiled through a literature search in Scopus and Web of Science using combinations of the search terms: (Lecithopyge OR Dolichosaccus OR Cephalogonimus OR Opisthioglyphe OR "Opisthioglyphe ranae" OR rastellus) AND (amphib* OR frog* OR salamander* OR newt* OR toad*). Publications were screened for further relevant citations. Both abstracts and full texts were reviewed to extract occurrence data.

| Host | Trivial name | Life stage | Site of infection | Description of trematode | Region | Referemce |
| --- | --- | --- | --- | --- | --- | --- |
| ***Lecithopyge* sp.** | | | | | | |
| Family Alytidae | | | | | | |
| *Alytes obstetricans* | Common midwife toad | – | – | – | France | [1] via [2] |
| *Discoglossus sardus* | Tyrrhenian painted frog | A | Intestine | *Opisthioglyphe rastellus* | France/ Corse | [3] |
| Family Bombinatoridae | | | | | | |
| *Bombina bombina* | European fire-bellied toad | – | – | – | Czech Republic/Slovakia | [4] via [2] |
|  |  | – | – | – | Czech Republic/Slovakia | [5] via [2] |
|  |  | – | – | – | Czech Republic/Slovakia | [6] via [2] |
| *Bombina variegata* | Yellow-bodied toad | – | – | – | Czech Republic/Slovakia | [7] via [2] |
|  |  | – | – | – | Czech Republic/Slovakia | [4] via [2] |
|  |  | – | – | – | Czech Republic/Slovakia | [8] via [2] |
|  |  | – | – | – | Czech Republic/Slovakia | [5] via [2] |
|  |  | – | – | – | Czech Republic/Slovakia | [9] via [2] |
|  |  | – | – | – | Czech Republic/Slovakia | [6] via [10] |
|  |  | A | Intestine | *Dolichosaccus rastellus* | Greece | [11] |
|  |  | A | Intestine | *Lecithopyge rastellus* | Ukraine | [12] |
| Family Bufonidae | | | | | | |
| *Bufo bufo* | Common toad | – | – | – | Denmark | [13] via [2] |
|  |  | – | – | – | England | [14] |
|  |  | A | Small intestine | *Dolichosaccus rastellus* | England | [15] |
|  |  | MC | Lung rudiments, small intestine wall, body tissues | *Dolichosaccus rastellus* | England | [15] |
|  |  | A | Intestine | *Distoma rastellus* | Sweden | [16] |
|  |  | – | – | – | Switzerland | [17] via [2] |
| *Bufotes viridis* | European green toad | – | – | – | Denmark | [13] via [2] |
| Family Ranidae | | | | | | |
| *Rana arvalis* | Moor frog | – | – | – | Czech Republic/Slovakia | [6] via [10] |
|  |  | – | – | – | Denmark | [13] via [2] |
|  |  | – | – | – | Russia | [18] via [10] |
|  |  | A | Intestine | *Dolichosaccus rastellus* | Russia | [19] |
|  |  | A | Small intestine | *Dolichosaccus rastellus* | Russia | [20] |
|  |  | A | Intestine | *Dolichosaccus rastellus* | Russia | [21] |
|  |  | – | – | *Dolichosaccus rastellus* | Sweden | [22] |
| *Rana macrocnemis* | Long-legged wood frog | A | Intestine | *Opisthioglyphe rastellus* | Georgia | [23] |
|  |  | A | Intestine | *Dolichosaccus rastellus* | Turkey | [10] |
| *Rana macrocnemis* (rec. as  *Rana camerani*) |  | A | Intestine | *Dolichosaccus rastellus* | Iran | [24] |
|  |  | A | Intestine | *Opisthioglyphe rastellus* | Turkey | [2] |
|  |  | A | Intestine | *Opisthioglyphe rastellus* | Turkey | [25] |
| *Rana graeca* | Greek stream frog | – | – | *Opisthioglyphe rastellus* | Bulgaria | [26] |
| *Rana holtzi* | Taurus frog | A | Intestine | *Opisthioglyphe rastellus* | Turkey | [27] |
| *Rana temporaria* | Common frog | – | – | *Opisthioglyphe rastellus* | Austria | [28] via [3] |
|  |  | – | – | – | Czech Republic/Slovakia | [5] via [2] |
|  |  | – | – | *Opisthioglyphe rastellus* | Czech Republic/Slovakia | [6] via [10] |
|  |  | A | Small intestine | *Opisthioglyphe rastellus* | Denmark | [29] |
|  |  | – | – | – | Denmark | [13] via [2] |
|  |  | – | – | – | England | [14] |
|  |  | A | Intestine | *Opisthioglyphe rastellus* | France | [30] |
|  |  | A | Intestine | *Opisthioglyphe rastellus* | France | [31] |
|  |  | – | – | – | Germany | [32] via [10] |
|  |  | – | – | *Opisthioglyphe rastellus* | Germany | [28] via [3] |
|  |  | A | – | *Dolichosaccus rastellus* | Ireland | [33] |
|  |  | – | – | – | Poland | [34] via [3] |
|  |  | A | Small intestine | *Dolichosaccus rastellus* | Russia | [35] |
|  |  | A | Intestine,  Oesophagus | *Distoma rastellus* | Sweden | [16] |
|  |  | – | – | *Dolichosaccus rastellus* | Sweden | [22] |
|  |  | – | – | *Opisthioglyphe rastellus* | Switzerland | [36] |
|  |  | A | Small intestine | *Dolichosaccus rastellus* | UK | [37] |
|  |  | – | – | – | UK | [38] via [10] |
|  |  | A | Small intestine | *Dolichosaccus rastellus* | UK | [39] |
|  |  | A | Small intestine | *Dolichosaccus rastellus* | UK | [40] |
|  |  | A | Small intestine | *Dolichosaccus rastellus* | UK | [41] |
|  |  | – | – | *Opisthioglyphe rastellus* | North Macedonia | [42] |
| *Rana amurensis* (rec. as *Rana amurensis asiatica)* | Khabarovsk frog/ Siberian wood frog | – | – | *Dolichosaccus rastellus* | Kyrgyzstan | [43] |
| *Pelophylax lessonae* | Pool frog | A | Small intestine | *Dolichosaccus rastellus* | Russia | [44] |
| *Pelophylax ridibundus* | Marsh frog | A | Intestine | *Opisthioglyphe rastellus* | Georgia | [23] |
|  |  | A | Small intestine | *Dolichosaccus rastellus* | Ukraine | [45] |
| *Pelophylax* kl. e*sculentus* (rec. as *Rana esculenta*) | Edible frog/ Common water frog | – | – | – | Czech Republic/Slovakia | [7] via [2] |
|  |  | – | – | – | Czech Republic/Slovakia | [4] via [2] |
|  |  | – | – | – | Denmark | [13] via [2] |
|  |  | – | – | – | UK | [38] via [10] |
|  |  | A | Small intestine | *Dolichosaccus rastellus* | UK | [41] |
| Family Salamandridae | | | | | | |
| *Ichthyosaura alpestris* (rec. as *Triturus alpestris*) | Alpine newt | J | Small intestine | *Dolichosaccus rastellus* | Greece | [11] |
|  |  | MC | Buccal cavity, gut wall |  |  |  |
| *Salamandra salamandra* (rec. as *Salamandra maculosa*) | Fire salamander | – | – | – | France | [1] via [2] |
|  |  | – | – | – | Switzerland | [17] via [2] |
| *Lissotriton helveticus* (rec. *as Triturus palmatus*) | Palmate newt | – | – | – | France | [1] via [2] |
| *Mertensiella caucasica* | Caucasian salamander | A | Intestine | *Opisthioglyphe rastellus* | Turkey | [46] |
| ***Cephalogonimus* sp.** | | | | | | |
| Family Ranidae | | | | | | |
| *Rana temporaria* | Common frog | – | – | – | Czech Republic/Slovakia | [6] via [10] |
|  |  | A | – | *Cephalogonimus europaeus* | France | [47] |
|  |  | MC | Mouth, gill arches |  |  |  |
|  |  | A | Intestine | *Cephalogonimus retusus* | UK | [41] |
| *Pelophylax ridibundus* | Marsh frog | – | – | – | Czech Republic/Slovakia | [6] via [10] |
|  |  | A | Small intestine | *Cephalogonimus retusus* | Georgia | [23] |
|  |  | A | Intestine | *Cephalogonimus retusus* | Turkey | [10] |
| *Pelophylax ridibundus* (rec. as *Rana ridibunda*) |  | – | – | – | Bulgaria | [48] |
|  |  | – | – | – | Bulgaria | [49] |
| *Pelophylax* kl. *esculentus* | Edible frog/ Common water frog | – | – | – | Czech Republic/Slovakia | [6] via [10] |
|  |  | – | – | – | Germany | [32] via [10] |
|  |  | – | – | – | Moldova | [50] via [10] |
|  |  | – | – | – | UK | [38] via [10] |
|  |  | A | Intestine | *Cephalogonimus retusus* | UK | [41] |
| *Pelophylax perezi* (rec. as *Rana perezi*) | Iberian waterfrog | – | – | *Cephalogonimus europaeus* | Spain | [51] |
| *Pelophylax perezi* (rec. as *Rana ridibunda perezi*) |  | – | – | *Cephalogonimus europaeus* | Spain | [52] |
| Family Pelobatidae | | | | | | |
| *Pelobates cultripes* | Western spadefoot | A | – | *Cephalogonimus europaeus* | France | [47] |
|  |  | MC | Mouth, gill arches |  |  |  |
| Family Colubridae | | | | | | |
| *Dolichophis jugularis* (rec. as *Coluber jugularis*) | Black whipsnake | – | – | – | Bulgaria | [53] via [10] |
| *Natrix natrix* | Grass snake | – | – | – | Bulgaria | [54] via [55] |
| *Natrix tessellata* | Dice snake | – | – | – | Bulgaria | [56] via [10] |
| Family Psammophiidae | | | | | | |
| *Malpolon monspessulanus* | Montpellier snake | – | – | – | Bulgaria | [53] via [10] |
| ***Opisthioglyphe ranae*** | | | | | | |
| Family Bombinatoridae | | | | | | |
| *Bombina bombina* | European fire-bellied toad | – | – | – | Czech Republic/Slovakia | [6] via [10] |
| *Bombina variegata* | Yellow-bodied toad | – | – | – | Czech Republic/Slovakia | [6] via [10] |
| Family Bufonidae | | | | | | |
| *Bufo bufo* | Common toad | – | – | – | Czech Republic/Slovakia | [6] via [10] |
|  |  | – | – | – | UK | [38] via [10] |
| *Bufo bufo* (rec. as *Bufo vulgaris*) |  | – | – | – | Germany | [32] via [10] |
| *Bufotes viridis* | European green toad | – | – | – | Czech Republic/Slovakia | [6] via [10] |
|  |  | – | – | – | Germany | [32] via [10] |
|  |  | – | – | – | Russia | [57] |
| *Epidalea calamita* (rec. as *Bufo calamita*) | Natterjack toad | – | – | – | Czech Republic/Slovakia | [6] via [10] |
|  |  | – | – | – | Germany | [32] via [10] |
|  |  | – | – | – | UK | [38] via [10] |
| Family Hylidae | | | | | | |
| *Hyla arborea* | European tree frog | – | – | – | Czech Republic/Slovakia | [6] via [10] |
| Family Ranidae | | | | | | |
| *Rana arvalis* | Moor frog | – | – | – | Czech Republic/Slovakia | [6] via [10] |
|  |  | A | Small intestine | *Opisthioglyphe ranae* | Kazakhstan | [58] |
|  |  | A | Small intestine | *Opisthioglyphe ranae* | Russia | [19] |
|  |  | A | Small intestine | *Opisthioglyphe ranae* | Russia | [20] |
|  |  | – | – | *Opisthioglyphe ranae* | Russia | [59] |
| *Rana dalmatina* | Agile frog | – | – | – | Czech Republic/Slovakia | [6] via [10] |
| *Rana temporaria* | Common frog | – | – | – | Czech Republic/Slovakia | [6] via [10] |
|  |  | – | – | – | Germany | [32] via [10] |
|  |  | MC | Oesophagus, head, spine, body | *Opisthioglyphe ranae* | Germany | [60] |
|  |  | – | – | – | UK | [38] via [10] |
|  |  | A | Small intestine | *Opisthioglyphe ranae* | Russia | [35] |
|  |  | – | – | – | Switzerland | [61] |
| *Pelophylax lessonae* | Pool frog | – | – | – | Czech Republic/Slovakia | [6] via [10] |
|  |  | A | Small intestine | *Opisthioglyphe ranae* | Poland | [62] |
|  |  | A | – | *Opisthioglyphe ranae* | Russia | [63] |
|  |  | – | – | *Opisthioglyphe ranae* | Russia | [59] |
| *Pelophylax ridibundus* | Marsh frog | – | – | – | Czech Republic/Slovakia | [6] via [10] |
|  |  | A | Small intestine | *Opisthioglyphe ranae* | Ukraine | [45] |
|  |  | – | – | *Opisthioglyphe ranae* | Russia | [59] |
| *Pelophylax ridibundus* (rec. as *Rana ridibunda*) |  | – | – | *Opisthioglyphe ranae* | Greece | [11] |
|  |  | A | Small intestine | *Opisthioglyphe ranae* | Iran | [64] |
|  |  | A | Small intestine | *Opisthioglyphe ranae* | Iraq | [65] |
|  |  | A | Small intestine | *Opisthioglyphe ranae* | Poland | [62] |
|  |  | A | Small intestine | *Opisthioglyphe ranae* | Turkey | [66] |
|  |  | A | Small intestine | *Opisthioglyphe ranae* | Turkey | [67] |
|  |  | – | – | *Opisthioglyphe ranae* | Turkey | [68] |
| *Pelophylax* kl. *esculentus* | Edible frog/ Common water frog | – | – | – | Czech Republic/Slovakia | [6] via [10] |
|  |  | – | – | – | Germany | [32] via [10] |
|  |  | – | – | – | Moldova | [50] via [10] |
|  |  | – | – | – | UK | [38] via [10] |
|  |  | A | Small intestine | *Opisthioglyphe ranae* | Ukraine | [45] |
|  |  | A | Small intestine | *Opisthioglyphe ranae* | Serbia | [69] |
|  |  | – | – | – | Switzerland | [61] |
|  |  | – | – | *Opisthioglyphe ranae* | Switzerland | [36] |
| *Pelophylax* kl. e*sculentus* (rec. as *Rana esculenta*) |  | A | Intestine | *Opisthioglyphe ranae* | Germany | [60] |
|  |  | MC | Oesophagus, head, spine, body |  |  |  |
|  |  | – | – | – | Russia | [70] |
| Family Pelobatidae | | | | | | |
| *Pelobates fuscus* | Common spadefoot | – | – | – | Russia | [18] via [10] |
| Family Salamandridae | | | | | | |
| *Triturus cristatus* (rec. as *Molge cristata*) | Northern crested newt | – | – | – | Germany | [32] via [10] |
| *Lissotriton vulgaris* (rec. as *Triturus palustris*) | Smooth newt | – | – | – | UK | [38] via [10] |
| Family Colubridae | | | | | | |
| *Natrix natrix* | Grass snake | – | – | *Opisthioglyphe ranae* | Belarus | [71] |
|  |  | – | – | – | Bulgaria | [54] via [55] |
|  |  | – | – | *Opisthioglyphe ranae* | Poland | [72] |

**References**

1. Joyeux C, Baer JG. Recherches sur le cycle évolutif de trématode *Opisthioglyphe rastellus* (Olsson, 1876). Bulletin biologique de la France et de la Begique. 1927:359–73.

2. Yildirimhan HS, Goldberg SR, Bursey CR. Helminth Parasites of the Banded Frog *Rana camerani* (Ranidae) from Turkey. Comp Parasitol. 2006; doi:10.1654/4229.1.

3. Combes C, Knoepffler LP. *Opisthioglyphe rastellus* (Olsson, 1876) (Trematoda, Digenea) chez *Discoglossus sardus* Tschudi, 1837, en Corse. Vie et Milieu. 1967:85–92.

4. Vojtková L. Larval stages of helminths. Ceskoslovenska Parasitologie. 1963:171–85.

5. Kozák A. Die Trematodenfauna des darpathengebietes der CSSR. Biologia (Bratislava). 1973:335–50.

6. Vojtková L, Votjek J. Die Trematoden der Amphibien in der Tschechoslowakei. II. Die Larvenstadien (Mesocercarien und Metacercarien). Folia Facultatis Scientiarum Naturalium Universitatis Purkynianae Brunensis Biologia. 1975:7–84.

7. Prokopic J. K helmintofaune nasich zab. Ceskoslovenska Parasitologie. 1957:249–62.

8. Vojtková L, Krivanec K. The helminth fauna of frogs from Moravia. Spisy prirodov Faculty Univiersy J. E. Plurkyne, Brno. 1970:253–82.

9. Prokopic J, Krivanec K. Helminths of amphibians, their interaction and host-parasite relationships. Acta Scientiarum Naturalium Brno. 1975:1–48.

10. Tepe Y, Yilan Y. New records of trematode and acanthocephalan species in frogs in Erzurum Province, Turkey. Helminthologia. 2021; doi:10.2478/helm-2021-0043.

11. Sattmann H. Endohelminths of some amphibians from Northern Greece (Trematoda, Acanthocephala, Nematoda; Amphibia: *Triturus, Rana, Bombina*). Herpetozoa. 1990:67–71.

12. Tkach V, Pawlowski J, Mariaux J. Phylogenetic analysis of the suborder plagiorchiata (Platyhelminthes, Digenea) based on partial lsrDNA sequences. Int J Parasitol. 2000; doi:10.1016/s0020-7519(99)00163-0.

13. Frandsen F. A study of Danish amphibians parasite fauna. Acta Parasitol Pol. 1974:49–66.

14. Loos A. Über einige zum Teil neue Distomen der europäischen Fauna. C. B. f. Bak. u. Parasit. Orig. 1907:604–13.

15. Diaz Diaz T. Studies on the life cycles of digenetic trematodes. PhD Thesis, University of Leeds, Leeds, West Yorkshire UK. 1976.

16. Olsson P. Bidrag till Skandinaviens Helminthfauna. Kongl Svenska Vetenskaps-Academiens Handlingar. 1876:1–35.

17. André E. Recherches parasitologiques sur les amphibiens de la Suisse. Revue Suisse de Zoologie. 1912:471–85.

18. Ruchin AB, Chikhlyaev IV, Lukyanov SV. [Analysis of helminthofauna of Common spaedfoot *Pelobates fuscus* (Laurenti, 1768) and moor frog *Rana arvalis* Nilsson, 1842 (Amphibia: Anura) at their joint habitation]. In Russian. Parazitologiia. 2009:240–7.

19. Zhigileva ON, Kirina IY. Helminth infestation of the moor frog (*Rana arvalis* Nilsson, 1842) and the Siberian tree frog (*Rana amurensis* Boulenger, 1886) in Western Siberia. Contemp Probl Ecol. 2015; doi:10.1134/S1995425515020171.

20. Chikhlyaev IV, Ruchin AB. An overview of the helminths of Moor Frog *Rana arvalis* Nilsson, 1842 (Amphibia: Anura) in the Volga Basin. Diversity. 2021; doi:10.3390/d13020061.

21. Burakova AV, Vershinin VL, Vershinina SD. Comparative analysis of the parasite fauna of *Rana arvalis* in the environmental gradients of Ural. Inland Water Biol. 2022; doi:10.1134/S1995082922040289.

22. Cedhagen T. Endoparasites in some Swedish amphibians. Acta Parasitol Pol. 1988;33:107–13.

23. Arabuli L, Murvanidze L, Faltynkova A, Mumladze L. Checklist of digeneans (Platyhelminthes, Trematoda, Digenea) of Georgia. Biodivers Data J. 2024; doi:10.3897/BDJ.12.e110201.

24. Mashaii N. New records of trematode parasites (Digenea) in the banded frog (*Rana camerani*) and marsh frog (*Rana ridibunda ridibunda*) (Anura: Ranidae), from Southwest of Iran. IJFS. 1999:41–8.

25. Düşen S. Helminths of the two mountain frogs, banded frog, *Rana camerani* boulenger, 1886 and Uludağ frog *Rana macrocnemis* boulenger, 1885 (Anura: Ranidae), collected from the Antalya province. Turkiye Parazitol Derg. 2007.

26. Bozhkov D, Stoikova P. Helminth fauna of *Rana graeca* in Bulgaria. Izvestiya na Zoologicheskiya Institut s Muzei. 1970:69–75.

27. Yildirimhan HS, Bursey CR, Goldberg SR. Helminth parasites of the Taurus Frog, *Rana holtzi*, and the Uludag Frog, *Rana macrocnemis*, with remarks on the helminth community of Turkish anurans. Comp Parasitol. 2006; doi:10.1654/4191.1.

28. Travassos L. Pesquizas helminthologicas realisadas em Hamburgo. Mem. Inst. Oswaldo Cruz. 1930;23:163–8.

29. Bovien P. Forelobig meddelelse om forekomst og hyppighed af trematoder hos *Rana esculenta* og *R. platyrrhinus*. Videnskabelige meddelelser fra Dansk naturhistorisk forening I kobenhavn. 1916:16–7.

30. Combes C. Biologie, écologie des cycles et biogéographie de digènes et monogènes d’Amphibiens dans l’Est des Pyrénées. Mémoires du Muséum National d’Histoire Naturelle. Série A, Zoologie. 1968.

31. Combes C, Leger N, Pesson B. Variations dans le temps des populations d’Helminthes parasites de *Rana temporaria.* [Variations in time of populations of helminth parasites of *Rana temporaria* L]. Ann Parasitol Hum Comp. 1971;46:685–98. doi:10.1051/parasite/1971466685.

32. Lühe M. Parasitische Plattwürmer. I: Trematodes. Süsswasserfauna Deutschlands. Eine Exkursionsfauna. 1909:1–217.

33. Griffin CT. *Oswaldocruzia filiformis* (Nematoda: Trichostrongyloidea) in frogs (*Rana temporaria*) from three locations in Ireland. J Helminthol. 1989; doi:10.1017/S0022149X00008737.

34. Sandner H. Contribution a la connaissance de la faune parasitaire des Batraciens des environs de Varsovie. Acta Zool. et Oecol. Univ. Lodz. 1949:1–28.

35. Chikhlyaev I, Ruchin A. The helminth fauna study of European common brown frog (*Rana temporaria* Linnaeus, 1758) in the Volga basin. Acta Parasitol. 2014; doi:10.2478/s11686-014-0268-5.

36. Gassmann M. A study of the Trematoda and Acanthocephala of amphibians in the Jura region. (Preliminary note). Revue Suisse de Zoologie. 1972;79:980–98.

37. Baylis HA. XLI.— Records of some parasitic worms from British vertebrates. Ann Mag Nat Hist. 1928;1:329–43.

38. Dawes B. The Trematoda. With special reference to British and other European forms. Cambridge Univ. Press. 1946:644 pp.

39. Lees E. The incidence of helminth parasites in a particular frog population. Parasitology. 1962; doi:10.1017/s0031182000024033.

40. Cox FEG. Parasites of British amphibians. J Biol Educ. 1971; doi:10.1080/00219266.1971.9653665.

41. Smyth JD, Smyth MM. Trematoda. In: Smyth JD, Smyth MM, editors. Frogs as host-parasite systems I. London: Palgram; 1980.

42. Hristovski ND, Lees E. The helminth fauna of *Rana temporaria* in relation to that of Europe generally. Acta Parasitologica Iugoslavica. 1973:93–8.

43. Sarbagisheva MS. The trematode fauna of frogs in the Issyk-kul basin. Trudy Zoologicheskogo Instituta, Leningrad (Issledovaniya po morfologii i faunistike paraziticheskikh cherveĭ). 1983:68–75.

44. Chikhlyaev IV, Ruchin AB, Fayzulin AI. Short communication: An overview of the trematodes fauna of pool frog *Pelophylax lessonae* (Camerano, 1882) in the Volga Basin, Russia: 1. Adult stages. Nusantara Biosci. 2018; doi:10.13057/nusbiosci/n100410.

45. Kuzmin Y, Dmytrieva I, Marushchak O, Morozov-Leonov S, Oskyrko O, Nekrasova O. Helminth Species and infracommunities in frogs *Pelophylax ridibundus* and *P. esculentus* (Amphibia: Ranidae) in Northern Ukraine. Acta Parasitol. 2020; doi:10.2478/s11686-019-00164-3.

46. Yildirimhan HS, Bursey CR, Goldberg SR. Helminth parasites of the Caucasian Salamander, *Mertensiella caucasica*, from Turkey. Comp Parasitol. 2005; doi:10.1654/4152.

47. Combes C, Coll AM. Life-cycle of *Cephalogonimus europaeus* Blaizot, 1910 (Trematode, Cephalogonimidae). Bulletin de la Societe Neuchateloise des Sciences Naturelles. 1974:203–14.

48. Olson PD, Cribb TH, Tkach VV, Bray RA, Littlewood DTJ. Phylogeny and classification of the Digenea (Platyhelminthes: Trematoda). Int J Parasitol. 2003; doi:10.1016/s0020-7519(03)00049-3.

49. Bray RA, Webster BL, Bartoli P, Littlewood DTJ. Relationships within the Acanthocolpidae Lühe, 1906 and their place among the Digenea. Acta Parasitol. 2005;50:281–91.

50. Erhan D, Gherasim E. Trematodofauna complexului *Pelophylax esculenta* (amphibia, anura) din Codrii Centrali ai Republicii Moldova. 1. Familiile Plagiorchiidae, Cephalogonimidae. Studia Universitatis Moldaviae. Real and Natural Sciences Series. 2015;71:148–59.

51. Lluch J, Roca V, Navarro P. A contribution to the knowledge of the helminth fauna of Spanish reptiles and amphibians. III. Digenea, Paramphistomidae, Hemiuridae, Gorgoderidae, Plagiorchiidae, Haematoloechidae and Cephalogonimidae of Rana perezi Seoane, 1985 (Amphibia: Ranidae). Revista Ibérica de Parasitologia. 1986;46:387–92.

52. Combes C, Sarrouy H. Helminths of *Rana ridibunda perezi* (Amphibia) in the Soria region. Revista Iberica de Parasitologia. 1971;31:115–9.

53. Kirin D. *Metaleptophallus gracillimus* (Lühe, 1909), (Family Plagiorchiidae, Lühe, 1901) and *Cephalogonimus retusus* (Dijardin, 1845), (Family Cephalogonimidae, Nicol, 1915), new species of the helminth fauna of the Reptiles (Reptilia) in Bulgaria. Trav. Sci. Univ. Plovdiv, Animalia. 1994;30:41–6.

54. Kirin D. New records of the helminth fauna from grass snake, *Natrix natrix* L., 1758 and dice snake, *Natrix tessellata* Laurenti, 1768 (Colubridae : Reptilia) in South Bulgaria. Acta Zool Bulg. 2002;54:49–53.

55. Yildirimhan HS, Bursey CR, Goldberg SR. Helminth parasites of the grass snake, *Natrix natrix*, and the dice snake, *Natrix tessellata* (Serpentes: Colubridae), from Turkey. Comp Parasitol. 2007; doi:10.1654/4285.1.

56. Buchvarov G, Kirin D, Kostadinova A. Platyhelminth parasite assemblages in two species of snakes *Natrix natrix* and *Natrix tessellata* (Reptilia, Colubridae) from Bulgaria: seasonal variation. J Environ Prot Ecol. 2000;1:124–31.

57. Chikhlyaev IV, Kirillova NY, Kirillov AA. Overview of helminths of amphibians (Amphibia) from the Samara region. Izvestiya of Samara Scientific Center of the Russian Academy of Sciences. 2018;20:385–400.

58. Tarasovskaja NE. The using of measurement analysis in the study of interspecific interactions between the helminthes of moor frog (*Rana arvalis*) in Pavlodar region. Biological Sciences of Kazakhstan. 2013;2(3).

59. Kirillova NY, Ruchin AB, Kirillov AA, Chikhlyaev IV, Alpeev MA. Overview of helminths in land vertebrates from the Mordovia Nature Reserve, European Russia. Nat Env Poll Tech. 2023; doi:10.46488/nept.2023.v22i04.001.

60. Andreas K. Helminthen einheimischer Froschlurche; 2007.

61. André E. Recherches parasitologiques sur les amphibiens de la Suisse II. Revue Suisse de Zoologie. 1913;21:79-200.

62. Popiołek M, Rozenblut-Kościsty B, Kot M, Nosal W, Ogielska M. Endoparasitic helminths of water frog complex in Poland: do differences exist between the parental species *Pelophylax ridibundus* and *Pelophylax lessonae*, and their natural hybrid *Pelophylax esculentus*? Helminthologia. 2011; doi:10.2478/s11687-011-0020-8.

63. Chikhlyaev IV, Ruchin AB. Ecological analysis and biodiversity of the helminth community of the pool frog *Pelophylax lessonae* (Amphibia: Anura) from floodplain and forest water bodies. Diversity. 2022; doi:10.3390/d14040247.

64. Mashaii N, Balouch M, Moubedi I. New records about helminth parasites of the marsh frog, *Rana ridibunda ridibunda* (Anura: Ranidae) from the north of Iran. Iranian J of Fisheries Science 2000. doi:10.22092/ijfs.2018.114858.

65. Saeed I, Al-Barwari SE, Al-Harmni K. I. A metazoan parasitological research of some Iraqi amphibians. Türkiye Parazitol Derg. 2007;31:337–45.

66. Düşen S, Öz M. Helminths of the marsh frog, *Rana ridibunda* Pallas, 1771 (Anura: Ranidae), from Antalya Province, Southwestern Turkey. Comp Parasitol. 2006; doi:10.1654/4162.1.

67. Düşen S, Oğuz M. Metazoan endoparasites of three species of anurans collected from the Middle Black Sea Region of Turkey. Helminthologia. 2010; doi:10.2478/s11687-010-0035-6.

68. Koyun M, Birlik S, Sümer N, Yildirimhan HS. Helminth fauna of Eurasian marsh frog, *Pelophylax ridibundus* (Pallas, 1771) (Anura: Ranidae) from Bingöl, Eastern Anatolia, Turkey. Biharean Biol. 2015;9:128–32.

69. Bjelić-Čabrilo O, Popović E, Paunović A. Helminthofauna of *Pelophylax* kl. *esculentus* (Linne, 1758) from Petrovaradinski Rit Marsh (Serbia). Helminthologia. 2009; doi:10.2478/s11687-009-0021-z.

70. Chikhlyaev IV, Fayzulin AI, Zamaletdinov RI. Data on the helminths of edible frog *Rana esculenta* Linnaeus, 1758 (Anura, Amphibia) in Middle-Volga region. Povolzhsky Ecol. J. 2009:270–4.

71. Shimalov VV, Shimalov VT. Helminth fauna of snakes (Reptilia, Serpentes) in Belorussian Polesye. Parasitology Research. 2000; doi:10.1007/s004360050055.

72. Kuśmierek N, Pyrka E, Popiołek M. Diversity of helminths in polish reptiles: a review. Biologia. 2020; doi:10.2478/s11756-019-00330-y.
